# Mutations of two *FERONIA-like receptor* genes enhance rice blast resistance without growth penalty

**DOI:** 10.1093/jxb/erz541

**Published:** 2020-01-27

**Authors:** Zhuhong Yang, Junjie Xing, Long Wang, Yue Liu, Jianing Qu, Yang Tan, Xiqin Fu, Qinlu Lin, Huafeng Deng, Feng Yu

**Affiliations:** 1 College of Biology, and Hunan Key Laboratory of Plant Functional Genomics and Developmental Regulation, Hunan University, Changsha, PR China; 2 State Key Laboratory of Hybrid Rice, Hunan Hybrid Rice Research Center, Hunan Academy of Agricultural Sciences, Changsha, PR China; 3 National Engineering Laboratory for Rice and By-product Deep Processing, Central South University of Forestry and Technology, Changsha, PR China; 4 The James Hutton Institute, UK

**Keywords:** FERONIA, FLR, *Magnaporthe oryzae*, rice, ROS), stress responses

## Abstract

Genes that provide resistance to fungi and/or bacteria usually reduce plant growth and ultimately affect grain yield. Thus, crop breeding programs need to find genetic resources that balance disease resistance with growth. The receptor kinase FERONIA regulates cell growth and survival in Arabidopsis. Here, we investigate, in rice, the role of members of the *FERONIA-like receptor* (FLR) gene family in the balance between growth and the response to the fungal pathogen *Magnaporthe oryzae* (*Pyricularia oryzae*), which causes the most devastating disease in rice. We carried out genome-wide gene expression and functional screenings in rice via a gene knockout strategy, and we successfully knocked out 14 *FLR* genes in rice. Using these genetic resources, we found that mutations in the *FLR2* and *FLR11* genes provide resistance to rice blast without a profound growth penalty. Detailed analyses revealed that *FLR2* mutation increased both defense-related gene expression and *M. oryzae-*triggered production of reactive oxygen species. Thus, our results highlight novel genetic tools for studying the underlying molecular mechanisms of enhancing disease resistance without growth penalty.

## Teaser

The FERONIA-like receptor (FLR) is essential for the response to *Magnaporthe oryzae* in rice, and mutations in *FLR2* and *FLR11* enhance resistance in the absence of significantly growth penalty.

## Introduction

Genetic immunity to disease is usually accompanied by unexpected reductions in growth and productivity (Ning *et al.*, 2017). Promising new crop varieties (for example in rice) should have a sufficient balance between plant disease resistance and economic characteristics. Thus, finding genetic resources that balance disease resistance with growth is needed for crop breeding.

Plants perceive and process external and internal information to form coordinated responses, which are critical to adapt to environmental conditions. Most of the information is processed by plasma membrane (PM)-localized receptor-like kinases (RLKs), which perceive signals via an N-terminal extracellular domain and activate downstream signaling cascades via a C-terminal intracellular kinase domain (Liao *et al.*, 2017; Franck *et al.*, 2018). Members of the RLK1-like (*Cr*RLK1Ls) subfamily, which are also known as malectin RLKs, were first discovered in *Catharanthus roseus* (Schulze-Muth *et al.*, 1996) and found to be conserved in green plants. Malectin, first described in *Xenopus laevis*, plays a role in recognizing Glc_2_-*N*-glycan (Schallus *et al.*, 2008). There are 17 *Cr*RLK1L members in Arabidopsis, each of which contains an extracellular malectin-like domain (MLD), a transmembrane domain, and a cytoplasmic kinase domain (Lindner *et al.*, 2012). FERONIA (FER), a member of the *Cr*RLK1L subfamily, and its relatives are promising candidates that are capable of balancing disease resistance with yield and growth. FER and other *Cr*RLK1L members play essential role in cell growth, seed yield, and stress responses in plants (Liao *et al.*, 2017; Franck *et al.*, 2018). *FER* loss-of-function mutants present distorted trichomes, relatively short root hairs (Duan *et al.*, 2010), and box-shaped epidermal cells (Li *et al.*, 2015) in Arabidopsis, and exhibit semi-dwarf plant height (Li *et al.*, 2016; Pu *et al.*, 2017) in rice. FER is involved in the crosstalk between several hormone pathways that regulate plant growth. For example, FER regulates auxin-stimulated root hair elongation (Duan *et al.*, 2010) and mediates the crosstalk between abscisic acid (ABA) and rapid alkalinization factor1 (RALF1, a ligand of FER) to modulate root growth under stress conditions (Yu *et al.*, 2012). FER has also been implicated in ethylene- (Mao *et al.*, 2015) and brassinosteroid-mediated (Guo *et al.*, 2009) responses that control cell growth. One of the downstream responses of the RALF1–FER pathway is the recruitment of RIPK, which inhibits H^+^ pump activity to increase the pH and inhibit cell growth in roots (Pearce *et al.*, 2001; Haruta et al., 2008, 2014; Du *et al.*, 2016). Recently, EBP1 was identified as a downstream factor in the RALF1–FER pathway and was shown to regulate nuclear gene expression and cell size (Li *et al.*, 2018). Relatives of the FER gene family also affect Arabidopsis growth; THESEUS1 (THE1) and HERCULES1 (HERK1) act redundantly to control cell elongation, as *the1* and *herk1* single mutants exhibit no defective vegetative phenotype, while the leaves and stems of *the1/herk1* double mutants and *the1/herk1/herk2* triple mutants exhibit cell elongation defects (Guo *et al.*, 2009). *ERULUS/[Ca*^*2+*^*]*_*cyt*_*-associated protein kinase* (*ERU/CAP1*) and *CURVY1* (*CVY1*) mutants have short root hairs and box-shaped pavement cells, respectively (Bai *et al.*, 2014; Gachomo *et al.*, 2014). ANXUR1 (ANX1) and ANX2 are preferentially expressed in pollen, and control pollen tube growth and rupture by interacting with BUDDHA PAPER SEAL1 (BUPS1) and BUPS2 via ectodomains and via the formation of complexes (Boisson-Dernier *et al.*, 2009; Miyazaki *et al.*, 2009; Ge *et al.*, 2017). During plant reproductive development, RALF4 and RALF19 directly interact with the ANX1/2–BUPS1/2 complex to regulate pollen tube integrity and growth, and RALF34 can subsequently replace RALF4 and RALF19 to bind with ANX1/2–BUPS1/2 and induce pollen tube rupture and sperm release when the pollen tube enters the ovule (Ge *et al.*, 2017; Mecchia *et al.*, 2017). In rice, the FER homologs FLR1 and FLR2 redundantly control plant height, tillering, branching, and fertility (Li *et al.*, 2016; Pu *et al.*, 2017); the last three are critical factors that control grain yield (Gravois and McNew, 1993). *Ruptured pollen tube* (*RUPO*), a relative of the FLR1/2 gene family, is expressed specifically in pollen and regulates pollen tube growth and integrity (Liu *et al.*, 2016), indicating that FER and its homologs are highly conserved signaling molecules that are involved in plant cell growth and yield.

FER also affects the immune response of Arabidopsis. *FER* mutations have conferred increased resistance to the pathogens *Golovinomyces* (syn. *Erysiphe*) *orontii* (Kessler *et al.*, 2010) and *Fusarium oxysporum* (Masachis *et al.*, 2016), but also increased susceptibility to *Hyaloperonospora arabidopsidis* and *Colletotrichum higginsianum* (Kessler *et al.*, 2010). Recently, FER was confirmed to be essential for pathogen-associated molecular pattern (PAMP)-triggered immunity (PTI). FER is required for flg22-, elf18-, and chitin-triggered reactive oxygen species (ROS) production. Moreover, Arabidopsis RALF23 inhibits plant immunity via recruiting FER and inhibiting pattern recognition receptor (PRR) complex formation (Stegmann *et al.*, 2017). Fungal RALF mimics have been identified in a wide range of plant pathogens and in some bacteria (Masachis *et al.*, 2016; Thynne *et al.*, 2017). The pathogenic fungus *F. oxysporum* can secrete RALF1-like proteins (F-RALFs), which can bind with FER to negatively regulate H^+^ pump activity and stimulate apoplastic alkalinization to suppress plant immunity and facilitate pathogenicity (Masachis *et al.*, 2016). FER can associate with FLS2 and its co-receptor BAK1 (Stegmann *et al.*, 2017), which can form complexes to detect flg22 and interfere with the assembly of FLS2–BAK1 complexes to modulate plant PTI signaling. Furthermore, ANX1 can associate with the leucine-rich repeat (NLR) proteins RPS2 and RPM1, which have a nucleotide-binding domain, to accelerate RPS2 degradation and inhibit RPS2-regulated cell death. Thus, ANX1 and ANX2 negatively control both plant PTI and effector-triggered immunity (ETI) (Mang *et al.*, 2017). THE1 can interact with GUANINE EXCHANGE FACTOR 4 (GEF4) to positively mediate defense responses against the necrotrophic fungus *Botrytis cinerea* (Qu *et al.*, 2017). However, another study verified that THE1 acts upstream of the mechanosensitive Ca^2+^ channel MATING PHEROMONE INDUCED DEATH 1 (MID1)-COMPLEMENTING ACTIVITY 1 (MCA1) and the RLK EFI2 to regulate cell wall integrity signaling, but is not required for PTI (Engelsdorf *et al.*, 2018).

Our previous study showed that FLR1 and FLR2 control reproductive development in rice (Li *et al.*, 2016), but the function of FLRs in vegetative growth and stress responses in rice is largely unknown. Here, we addressed this unknown by functionally characterizing the FLR gene family in rice.

## Materials and methods

### Identification of *FLR* genes in rice

The genome, transcript, DNA coding sequence (CDS), and peptide sequence data of wild rice (*Oryza rufipogon*) and *Oryza sativa indica* were downloaded from the Ensembl Plants database (http://plants.ensembl.org/index.html). The genomic data of *O. sativa japonica* Nipponbare (Nip) were downloaded from the Rice Genome Annotation Project database (RGAP; http://rice.plantbiology.msu.edu/). Seventeen Arabidopsis *Cr*RLK1L (including FER and its relatives) protein sequences (Lindner *et al.*, 2012) were downloaded from TAIR (ftp://ftp.arabidopsis.org). We used two methods to identify potential *Cr*RLK1L genes in rice: (i) the sequences of the MLD PF12819 and the Pkinase domain PF07714 were downloaded from the Pfam website (https://pfam.xfam.org) and used as queries to scan rice protein databases with HMMER version 3.0; and (ii) 17 Arabidopsis *Cr*RLK1L protein sequences were used as queries for the Basic Local Alignment Search Tool (BLAST) for searching against rice protein databases for potential *Cr*RLK1L genes. The candidate sequences were subsequently confirmed by the Conserved Domain Database (CDD) tool within the NCBI (https://www.ncbi.nlm.nih.gov/cdd) database. Finally, each putative *Cr*RLK1L protein sequence was manually examined via the Simple Modular Architecture Research Tool (SMART; http://smart.embl-heidelberg.de/) to ensure that it contained both the conserved MLD and Pkinase domain.

### Phylogenetic tree construction and motif composition analysis

Five *Physcomitrella patens Cr*RLK1L protein sequences (Rensing *et al.*, 2008; Lehti-Shiu and Shiu, 2012) and two *Selaginella moellendorffii Cr*RLK1L protein sequences (Banks *et al.*, 2011; Lehti-Shiu and Shiu, 2012) were downloaded from Phytozome version 7.0 (https://phytozome.jgi.doe.gov/). *Cr*RLK1L protein sequences were aligned via ClustalW software, with the default parameters (http://www.clustal.org/clustal2/), and a phylogenetic tree based on the Neighbor–Joining (NJ) method was constructed using MEGA 7.0 in accordance with the following parameters: 1000 bootstrap replications, the p-distance model, uniform rates among sites, and partial deletion (95% site coverage) for gaps and missing data. The phylogenetic tree was then optimized via iTOL (https://itol.embl.de/). Motifs were subsequently constructed via MEME (http://meme-suite.org/) by the use of the complete amino acid sequences of *Cr*RLK1L in accordance with the following parameters: number of repetitions, any; maximum number of motifs, 20; and optimum width of each motif, 6–50 residues (Bailey *et al.*, 2009).

### Gene sequence analysis of *FLR* genes in rice

The chromosome positions of the *FLR* genes were confirmed by the rice gene annotation gff3 file downloaded from the RGAP database (http://rice.plantbiology.msu.edu/), and the *FLR* genes were mapped to *O. sativa japonica* chromosomes via Map Gene 2 Chromosome V2 (MG2C; http://mg2c.iask.in/mg2c_v2.0/). The exon–intron structures of the *FLR* genes were analyzed by the Gene Structure Display Server (GSDS; http://gsds.cbi.pku.edu.cn/), and the Multiple Collinearity Scan toolkit (MCScanX) package (Wang *et al.*, 2012) was used to analyze the gene duplication events, with the default parameters. The syntenic relationships within the *FLR* family were displayed using Circos (Krzywinski *et al.*, 2009).

### Subcellular localization assays

*FLR3*, *-8*, *-11*, and *-14* were inserted into *pCAMBIA1300-GFP* vectors. The constructs were then introduced into Arabidopsis protoplasts via polyethyleneglycol (PEG)-mediated transformation as previously described (Li *et al.*, 2016). The protoplasts were observed via a Nikon confocal laser scanning microscope at 12–24 h after transformation.

### RNA isolation and gene expression analysis

For gene expression analysis, the roots, leaf sheaths, and leaves of Nip seedlings were harvested at the three-leaf stage. Mature roots, stems, flag leaves, panicles, and developing seeds were collected after the plants were grown in a greenhouse for 3–4 months. For temperature stress analysis, seedlings at the three-leaf stage were incubated at 4 °C for cold treatment and at 42 °C for heat treatment for 12 h; 25 °C was used as a control. To induce salt stress, the roots of seedlings at the three-leaf stage were immersed in Yoshida nutrient solution supplemented with NaCl (200 mM) for 3 d, and roots immersed in unaltered Yoshida nutrient solution were used as the control. After treatment, the shoots from the control and stressed seedlings were harvested for gene expression analysis. For biotic stress, Nip seedlings were sprayed with 70-15 spores as described previously (Xing *et al.*, 2017), and seedlings sprayed with 0.025% Tween-20 were used as the control. The inoculated and control plants were kept in a moist chamber for 24 h in the dark at 28 °C. Considering that all the aerial parts of seedlings could be infected, the shoots from the control and stressed seedlings were harvested, instantly frozen in liquid nitrogen, and then stored at –80 °C for gene expression analysis.

Total RNA extraction, reverse transcription, and quantitative real-time PCR (qRT-PCR) were performed as previously described (Li *et al.*, 2016). PCR was performed in triplicate for expression analysis. All the qRT-PCR data were quantified by the 2^–ΔΔCT^ method, with three technical replicates for each of the three biological replicates. The housekeeping gene *OsActin* (LOC_Os03g50885) was used as an internal control. The heatmaps were created by R version 3.5.1 with the pheatmap package on the basis of the log_2_-fold-transformed data. The primers used for qRT-PCR are listed in Table S1 at *JXB* online.

### Plant materials and transformation

T-DNA insertion mutants of *flr1* (Dongjin, DJ), *flr2* (Hwayoung, HY) (Li *et al.*, 2016), and *flr4* (DJ, PFG_1B-08401. R) were obtained from the Salk Institute (http://signal.salk.edu/cgi-bin/RiceGE). For clustered regularly interspaced short palindromic repeat (CRISPR)/CRISPR-associated protein 9 (Cas9) experiments, the guide RNA (gRNAs) targets in the *FLR* family gene and primers were selected via the E-CRISP Design Tool (http://www.e-crisp.org) (Heigwer *et al.*, 2014) and the CRISPR-GE tool (http://skl.scau.edu.cn) (Xie *et al.*, 2017). The Cas9 expression vectors were constructed as described previously (Ma *et al.*, 2015). The 20 bp target sequences were inserted into a pYLgRNA-OsU3 vector between the OsU3 promoter and the gRNA scaffolds, followed by ligation to the Cas9 expression backbone pYLCRISPR/Cas9Pubi-H vector. The sequence of the resulting vector, pYLCRISPR/Cas9Pubi-H, was verified and it was introduced into *Agrobacterium tumefaciens* EHA105 to infect embryogenic calli of wild-type Nip rice as previously described (Nishimura *et al.*, 2006). The transgenic rice lines were subsequently confirmed by sequencing PCR products that were amplified with primer pairs that flanked the designated target sites. Off-target sites were predicted by the CRISPR-GE tool (Xie *et al.*, 2017). The highest scoring off-target site was amplified from the genomic DNA of the mutated transgenic rice lines, and the resulting PCR products were analyzed by sequencing. At least two independent Cas9 knockout mutant lines were obtained for each gene, which were used for subsequent phenotypic assays.

To generate *FLR2*-overexpression (OE) lines, a primer pair was designed on the basis of the full-length CDS without a stop codon. The product of the CDS fragment was cloned into a *pCAMBIA1300-GFP* vector between the *Bam*HI and *Xba*I sites. After they were validated by sequencing, the constructs were transferred into rice embryogenic calli via the *Agrobacterium* strain EHA105 to generate the *FLR2*-OE lines. The gene expression levels of the *FLR2*-OE transgenic lines were determined by qRT-PCR at the RNA level and by western blot at the protein level. The transgenic and wild-type rice plants were grown in a greenhouse for 2 weeks and then transplanted to a field in Taohua village, Changsha, China (28°11'N, 112°58'E).

### *M. oryzae* isolates

The following *M. oryzae* isolates were used for inoculation: 70-15, which is compatible with Nip (Kim *et al.*, 2001) and is popular at many institutions; green fluorescent protein (GFP)-tagged 70-15 (70-15GFP); Guy11, which was generously provided by Dr Xuewei Chen (Sichuan Agricultural University); and HNB7, HNB16, HNB52, HNB62, HNB119, HNB145, and HNB154, which were collected from Hunan Province (Xing *et al.*, 2017). All strains were cultured on agar plates that contained complete medium (CM) under a 12 h light (by white fluorescent bulbs)/12 h dark photoperiod at 25 °C.

### Protein extraction and immunoblot analysis

For total protein extraction, fresh leaf tissues (0.1 g) were ground in liquid nitrogen and then incubated in 200 μl of protein extraction buffer [100 mM Tris–HCl, pH 7.5, 300 mM NaCl, 1 mM EDTA, 5% glycerol, 1% Triton X-100, 1 mM phenylmethylsulfonyl fluoride (PMSF), 1:100 protease inhibitor cocktail (78430, Thermo Fisher Scientific)] at 4 °C for 1 h. Proteins were separated in a 10% SDS–PAGE gel and subsequently transferred to polyvinylidene difluoride (PVDF) membranes. Anti-GFP (AT0028, CMC) and anti-actin (AT0004, CMC) antibodies were used for immunoblotting analysis.

### Pathogenicity assays

Conidia were harvested from 7- to 10-day-old cultures on complete agar medium supplemented with 0.025% Tween-20. The conidial suspension was adjusted to 1–1.5×10^5^ conidia ml^–1^ before wound inoculation or spray inoculation. Wound inoculation was performed as previously described (Zhang *et al.*, 2014), and inoculated plants were kept in a moist chamber for 24 h in the dark at 28 °C. The photoperiod was then adjusted to a 12 h light/12 h dark cycle for 5–7 d. The percentage of lesion areas (disease index) per leaf was scored via image analysis with ImageJ software. The relative fungal biomass was measured as previously described (Li *et al.*, 2017), and DNA-based quantitative PCR (qPCR) was performed with reference to the cycle threshold value of the *M. oryzae Mo*Pot2 gene and the rice genomic ubiquitin (*Os*Ubi) gene with the formula 2^[Ct(*Os*Ubi)–Ct(*Mo*Pot2)]^. The primers used for qRT-PCR are listed in Supplementary Table S1.

To observe fungal blast infection, detached leaf sheaths from the fourth leaf of five-leaf-stage seedlings were inoculated with the 70-15GFP isolate in a spore suspension (1×10^5^ conidia ml^–1^). The detached rice sheath inner epidermal sections were previously sliced to make temporary slides. The fluorescence signals of the conidia, conidia germination, appressorium development, and invasive hyphal growth in epidermal cells were observed and counted under a confocal microscope as previously described (Li *et al.*, 2017). For the percentage of *M. oryzae* hyphae in each type/stage, 50 hyphae were evaluated.

For field tests, the seedlings of the tested mutants and transgenic lines were cultivated in a greenhouse for 2 weeks before being transplanted into the field at the Daweishan blast nursery (Hunan Province, 28°45'N, 114°01'E) for resistance identification. When the seedlings were transplanted into the field, the planting area of each mutant and transgenic line was 10 m^2^, comprising a total of four rows and 25 cm×25 cm planting space. A row of Lijiangxintuanheigu (LTH), a highly susceptible variety, was planted between and around each variety used as an inducer to ensure uniform blast infection. Normal water and fertilizer management were applied, and fungicides were not applied throughout the whole growth period. The whole identification test was completely induced under natural conditions, with no artificial inoculation. Three months later, flag leaves and the second leaf above them were harvested to analyze relative fungal biomass.

### H_2_O_2_ accumulation

To visualize hydrogen peroxide (H_2_O_2_), 3,3-diaminobenzidine (DAB) staining was performed as described previously (Thordal-Christensen *et al.*, 1997), with slight modifications. Seedlings at the five-leaf stage were sprayed with the *M. oryzae* isolate in a spore suspension (1×10^5^ conidia ml^–1^). At 3 days post-inoculation (dpi), leaf sections were vacuum infiltrated with DAB solution [1 mg ml^–1^ DAB, 50 mM Tris–HCl, 0.01% Triton X-100, pH 6.5] for 10 min, after which the sections were incubated at 25 °C for 12 h in the dark. The DAB-stained leaves were cleared by boiling in 90% ethanol for 20 min and then observed under a microscope. The relative amount of H_2_O_2_ was calculated on the basis of the pixels of images via Photoshop with the following formula: H_2_O_2_ area per rectangle=pixels of H_2_O_2_ area per mycelial invasion site/pixels of the rectangle.

## Results

### Identification and phylogenetic analysis of *FLR* family *Cr*RLK1L homologs in rice

**Fig. 1.**
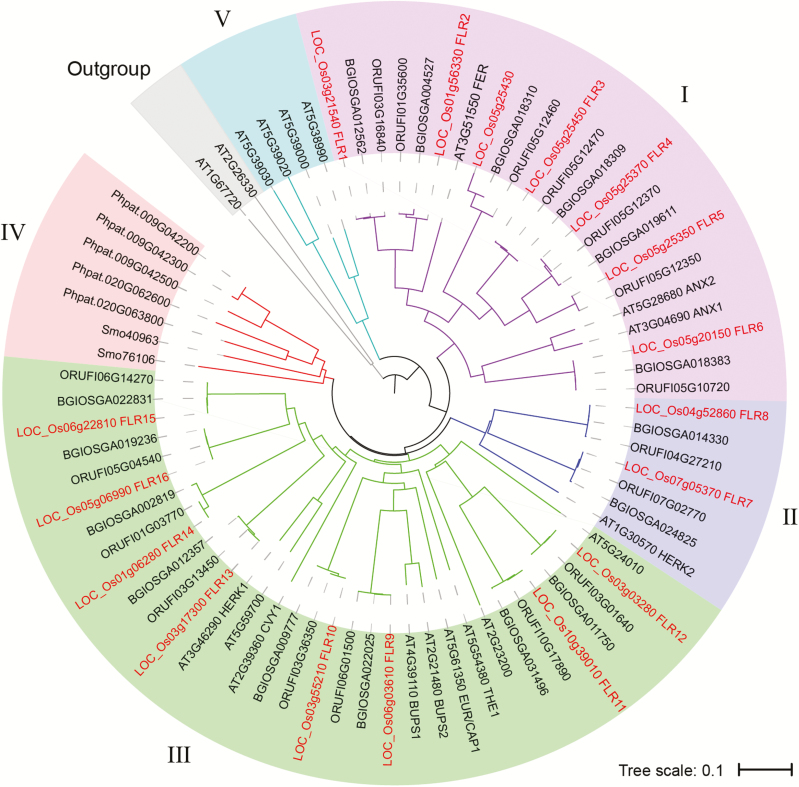
Phylogenetic tree of *Cr*RLK1L family proteins. Complete amino acid sequences were aligned using ClustalW. The phylogenetic tree was constructed via the NJ method with 1000 bootstrap values in MEGA7 and was optimized with the iTOL online tool. The analyzed *Cr*RLK1Ls were distributed in five main subgroups: I–V, marked with different background colors. Smo, *Selaginella moellendorffii*; Phpat, *Physcomitrella patens*; AT, *Arabidopsis thaliana;* ORUFI, *Oryza rufipogon*; BGIOSGA, Beijing Genomics Institute *Oryza sativa* Genome Annotation (*indica*); LOC_Os, *Oryza sativa loc*us (*japonica*). The *Cr*RLK1Ls (FLRs) of *Oryza sativa japonica* rice are highlighted in red.

The two main rice varietal groups, *O. sativa indica* and *O. sativa japonica*, were independently domesticated from *O. rufipogon* wild rice populations (Londo *et al.*, 2006; Huang *et al.*, 2012). To investigate *CrRLK1L*/*FLR* (named *FLR* in rice hereafter) homologous genes in rice, we performed a homology search via BLAST (see the Materials and methods), and we identified 17, 16, and 17 potential *FLR* genes within the genome of *O. rufipogon*, *O. sativa indica*, and *O. sativa japonica*, respectively. To discover the evolutionary origin of the putative members of the *Cr*RLK1L family, in which *FLR* genes in rice are included, 74 *Cr*RLK1L genes [five *P. patens*, two *S. moellendorffii*, 17 Arabidopsis, 17 *O. rufipogon*, 16 *O. sativa indica*, and 17 *O. sativa japonica Cr*RLK1L protein sequences, and an additional two outgroup protein sequences (AT1G67720 and AT2G26330) from Arabidopsis] were utilized to construct a phylogenetic tree via the NJ method. The results (Fig. 1) indicated that these 74 *Cr*RLK1L genes could be divided into five subgroups (subgroups I–V), of which there were 23 members in group I, seven members in group II, 33 members in group III, seven members in group IV, and four members in group V. Subgroups I, II, and III included at least one rice *Cr*RLK1L member and one Arabidopsis *Cr*RLK1L member, suggesting that the divergence and emergence of the *Cr*RLK1L family occurred early in the separation of rice and Arabidopsis. There was a one-to-one relationship between the *CrRLK1L* genes of wild rice and those of *O. sativa japonica*, while *O. sativa indica* rice lacked a member corresponding to wild rice ORUFI05G12350 and *O. sativa japonica* LOC_Os05g25350. No homologs had a similar full-length sequence to that of ORUFI05G12350/LOC_Os05g25350 while containing both the MLD PF12819 and Pkinase domain PF07714 (see the Materials and methods). These results demonstrated that rice *CrRLK1L* genes were conserved but were slightly divergent in terms of their evolution. Seven *O. sativa japonica* members in subgroup I were homologous to FER, ANX1, and ANX2, which implies that these *Cr*RLK1Ls may have a role in mediating pollen tube growth and fertilization. Subgroup IV contained *CrRLK1L* members of the seedless land plants *P. patens* and *S. moellendorffii*; these members were homologous to THE1, HERK1, and HERK2, which implies that these *Cr*RLK1Ls may have a similar role in maintaining cell wall integrity and cell growth. Subgroup V contained only Arabidopsis proteins (Fig. 1). The phylogenetic tree based only on the 74 *Cr*RLK1L gene kinase domains (Supplementary Fig. S1) showed a similar division to that in Fig. 1, except for subgroup II, which contained 24 members (five *P. patens*, two *S. moellendorffii*, five *Arabidopsis*, four *O. rufipogon*, four *O. sativa indica*, and four *O. sativa japonica CrRLK1L* genes).

### Multiple sequence alignment and motif analysis of *Cr*RLK1L proteins

We analyzed the domain conservation by performing a motif analysis using the MEME tool. We identified 20 conserved motif sequences from all of the *Cr*RLK1L proteins. As shown in Supplementary Fig. S2, all *Cr*RLK1L proteins had a similar motif composition with similar motif arrangements. Most members of the same subgroup had the same motifs; for instance, LOC_Os01g56330, LOC_Os03g21540, and AT3G51550 shared the same motifs. Motifs 13 and 19 were present within N-terminal signal peptides. Nine conserved motifs (motifs 6, 8, 10, 11, 14, 15, 16, 17, and 18) were distributed within the malectin domain region. The remaining nine motifs (motifs 1, 2, 3, 4, 5, 7, 9, 12, and 20) were distributed within the C-terminal kinase domain region. Notably, three proteins (AT5G39020, AT5G39030, and LOC_Os05g25430) had lost more than half of the conserved motifs within their C-terminal kinase domain region. All *Cr*RLK1L proteins were similar in length except for LOC_Os05g25430 of *O. sativa japonica*, which had a relatively short protein length; thus, LOC_Os05g25430 was excluded as a canonical *Cr*RLK1L member. The remaining *Cr*RLK1L members of *O. sativa japonica* were denoted as FLR1–FLR16 (Fig. 1), corresponding to the distribution of their phylogenetic relationships with FER. We focused on the role of these 16 members (Supplementary Table S2) in the balance between growth and the stress response in subsequent experiments.

### Gene structure, chromosomal distribution, and duplication in the FLR family

**Fig. 2.**
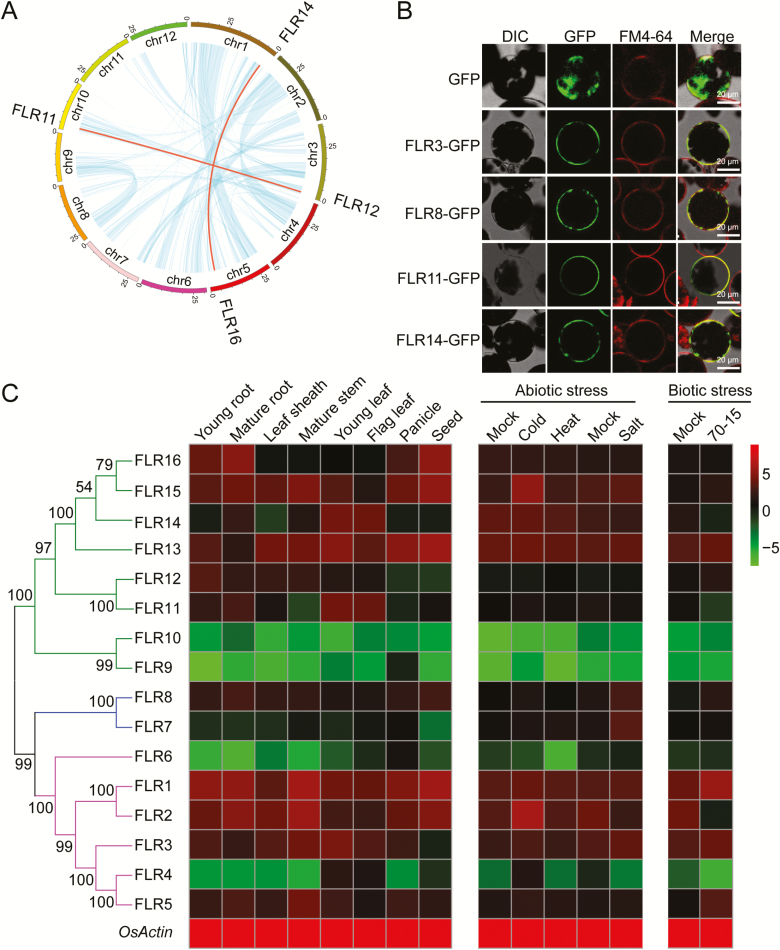
Analysis of duplication, subcellular localization, and gene expression in the rice FLR family. (A) Synteny analysis of *FLR* genes in *Oryza sativa japonica* rice. Chromosome numbers are shown on the inner side, and the blue lines show all synteny blocks in the rice genome. FLR gene pairs with syntenic relationships are linked by red lines. (B) The subcellular localization of FLR3–GFP, FLR8–GFP, FLR11–GFP, and FLR14–GFP in Arabidopsis protoplasts. (C) Expression patterns of *FLR* genes in different organs/stages and under different abiotic and biotic stresses (*n*=3 for each group). These experiments were performed three times, each yielding similar results.

The exon–intron structures of the *FLR* genes were examined to understand the gene structure evolution of the *FLR* family (Supplementary Fig. S3). All *FLR* genes had similar coding sequence lengths, and the vast majority of the *FLR* genes had no introns, except *FLR13* and *FLR15*, each of which contained one intron (Supplementary Fig. S3). Sixteen *FLR* genes were unevenly distributed among the 12 rice chromosomes (Supplementary Fig. S4), with the exception of chromosomes 2, 8, 9, 11, and 12. Chromosomes 3 and 5 contained relatively more *FLR* genes, with four and five genes, respectively, than did chromosomes 1 and 6, each of which contained two *FLR* genes. Chromosomes 4, 7, and 10 contained only one *FLR* gene each. Genomic duplications are considered an essential method to expand gene families and to increase the likelihood of evolutionary diversification. Ancient whole-genome duplication, chromosomal segmental duplication, and tandem duplication are the main dramatic forms of genomic duplication (Epstein, 1971; Lynch and Conery, 2000). In this study, we identified tandem and segmental duplication events in the FLR family. In accordance with the criterion that a chromosomal region of 200 kb containing two genes is defined as a tandem duplication event, three *FLR* genes (*FLR3*, *-4*, and *-5*) were clustered into such an event on chromosome 5 (Supplementary Fig. S3) (Holub, 2001). Another two segmental duplication events (*FLR11* to *FLR12* and *FLR14* to *FLR16*) among the 16 *FLR* genes were also identified by MCScanX (Fig. 2A). These results suggest that gene duplication occurred in the FLR family. The duplicated genes may have expanded the function of the family members to adapt to a more complex environment during the evolution of rice.

### Subcellular localization of four FLR family members

We previously reported that FLR1 and FLR2 localized to the PM (Li *et al.*, 2016) and are the closest homolog to FER in Arabidopsis. To determine the subcellular localization of FLR3, -8, -11, and -14, which form different subgroups (Fig. 1), we constructed a GFP fusion protein with a *35S* promoter and transiently expressed the construct in Arabidopsis protoplasts. The GFP fluorescence of FLR3–GFP, FLR8–GFP, FLR11–GFP, and FLR14–GFP was detected on the PM (indicated by FM4-64 membrane staining) of Arabidopsis protoplasts (Fig. 2B). These results are consistent with the conclusion that *Cr*RLK1L proteins act as sensors located on the cell membrane to perceive external signals and phosphorylate downstream proteins (Stegmann *et al.*, 2017).

### Expression patterns of *FLR* genes under normal growth and stress conditions

We analyzed the expression patterns of *FLR* family genes in different organs/stages and under various abiotic and biotic stresses via qRT-PCR. To compare the expression patterns, we converted the qRT-PCR gene expression values into a heatmap (Fig. 2C). Under normal growth conditions, *FLR1*, *-2*, and *-3* were relatively highly expressed in all tissues, while the expression levels of *FLR6*, *FLR9/RUPO*, and *FLR10* were extremely low in most tested tissues. In the vegetative organs, *FLR1*, *-2*, *-15*, and *-16* were highly expressed in the young roots and mature roots, and *FLR1*, -*2*, *-3*, *-5*, *-13*, and *-15* were highly expressed in the leaf sheaths and mature stems. *FLR1*, *-3*, *-11*, *-13*, and *-14* were highly expressed in the leaves and flag leaves. In the reproductive organs, *FLR1*, *-2*, *-13*, and *-15* were relatively abundant in the panicles, whereas *FLR1*, *-2*, -*13*, *-15*, and *-16* were predominantly highly expressed in developing seeds. Remarkably, *FLR9/RUPO* tended to be expressed in the panicles and, according to previous reports, *FLR9/RUPO* functions in pollen tube growth and integrity (Liu *et al.*, 2016)

Plants adapt to environmental changes and microbial attack by regulating gene expression. Under abiotic stress, compared with those in the control treatment, the expression levels of *FLR2*, *-4*, and *-15* in the cold treatment were up-regulated by >2-fold. Under high-temperature conditions, the expression of *FLR6* significantly decreased by 10-fold. Salinity stress caused a multifold up-regulation in the expression of *FLR7* and -*8*, while the expression levels of *FLR2* and *-4* were 2-fold down-regulated. To analyze the response of *FLR* genes to biotic stress, Nip seedlings were sprayed with the compatible *M. oryzae* strain 70-15. The transcription of *FLR2*, -*4*, -*11*, and -*14* was down-regulated by >2-fold after fungal treatment, while the expression of *FLR1*, *-5*, and *-8* greatly increased in response to this fungal attack (Fig. 2C).

### Screening the *FLR* gene family-mediated response to *M. oryzae*

To determine whether *FLR* family members function in rice immunity, we analyzed the function of *FLR* genes in response to *M. oryzae*. CRISPR/Cas9 technology was used to mutate 12 *FLR* family genes (Supplementary Fig. S5) in Nip; 11 (*FLR3*, *-5*, *-7*, *-8*, *-10*, *-11*, *-12*, *-13*, *-14*, *-15*, and *-16*) homozygous mutants were obtained, with at least two independent lines obtained for each gene. *FLR9/RUPO* mutants were heterozygous, which was in accordance with previous reports (Liu *et al.*, 2016). Moreover, we obtained three T-DNA insertion mutants: one was *FLR4* (DJ) from RiceGE (Supplementary Fig. S5), and the other two were *FLR1* (DJ) and *FLR2* (HY) mutants, which were identified in our previous works (Yang *et al.*, 2015; Li *et al.*, 2016).

**Fig. 3.**
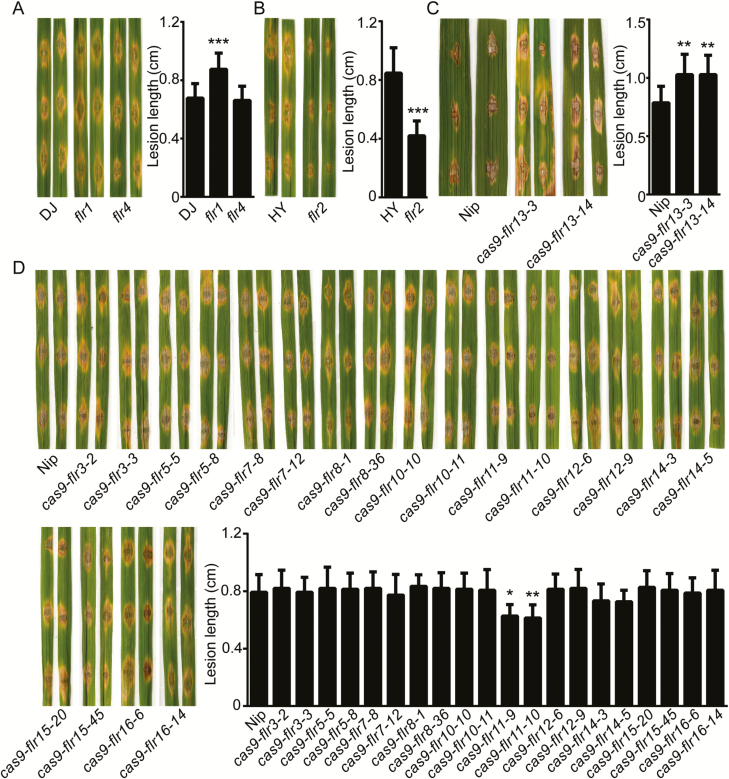
Function of the *FLR* gene family members in rice blast resistance. (A–D) Disease symptoms and lesion lengths of *FLR* mutant plants and wild-type plants at 7 dpi; the plants were inoculated (by wounding) with the compatible *Magnaporthe oryzae* isolate 70-15. (A) Disease symptoms and lesion lengths of *flr1* and *flr4* mutants and wild-type DJ plants. (B) Disease symptoms and lesion lengths of *flr2* and wild-type HY. (C and D) Disease symptoms and lesion lengths of 11 (*FLR3*, *-5*, *-7*, *-8*, *-10*, *-11*, *-12*, *-13*, *-14*, *-15*, and *-16*) mutants and wild-type Nip plants. (A–D) Statistical analysis of average lesion lengths, measured for five leaves (*n*=15 lesions for each group). The error bars correspond to 1 SD as determined by the duplicate analyses. The data are shown as the mean ±SD. The asterisks indicate significant differences from the control, as determined by a *t*-test or one-way ANOVA followed by Tukey’s test (*P<0.05, **P<0.01, ***P<0.001). These experiments were performed with three independent biological replicates, each yielding similar results, while wound inoculation of *flr13* with the rice blast fungus had only technical replicates, as *flr13* is sterile. (This figure is available in color at *JXB* online.)

To analyze the variation in *FLR* family mutations in response to rice blast fungus via these *FLR* family mutants, the leaves of the mutants were inoculated with *M. oryzae* by the wound method (Zhang *et al.*, 2014). As shown in Fig. 3, the lesion lengths of *flr1* and *flr13* were significantly longer than those of the wild-type control. These results suggest that *FLR1* and *FLR13* may be related to rice immunity. Nevertheless, the lesions of the *flr2 and flr11* mutants were significantly shorter and smaller than those of the wild-type HY and Nip plants, respectively. *flr14* was slightly resistant to *M. oryzae*, but its resistance was not significantly different from the control. The basal resistance of the transgenic plants of nine other genes (*FLR3*, *-4*, *-5*, *-7*, *-8*, *-10*, *-12*, *-15*, and *-16*) was similar to that of the wild-type plants, indicating that the loss of these genes did not cause a defect in rice blast fungus disease resistance. A similar conclusion was confirmed in two independent CRISPR/Cas9 lines with mutations in FLR family members. These results suggest that *FLR2* and *FLR11* negatively regulate rice blast fungus response. FLR2 was chosen for further analysis because it has a very high similarity to FER, which plays important roles in both growth and biotic stress responses (Stegmann *et al.*, 2017).

### *FLR2* negatively regulates rice resistance to *M. oryzae*

**Fig. 4.**
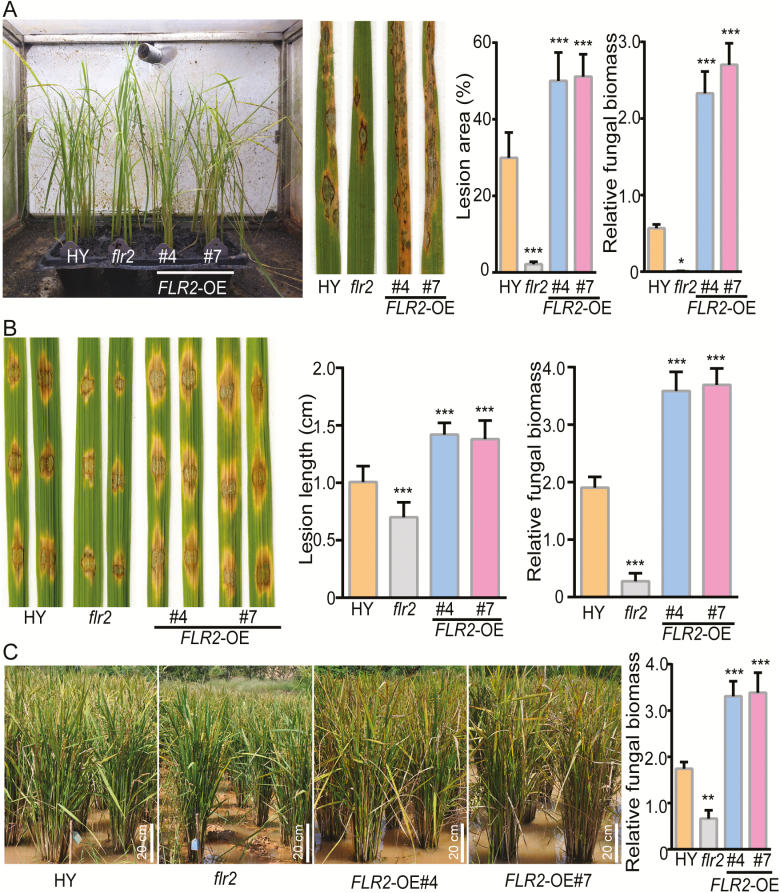
FLR2 functions in rice blast resistance. (A) Disease symptoms of HY, *flr2*, and *FLR2*-OE plants at 7 dpi; the plants were inoculated (by spraying) with the compatible *M. oryzae* isolate 70-15. The percentage of lesion areas (disease index) was scored via image analysis with ImageJ software (*n*=5 leaves). Fungal growth was measured by the expression level of the *Magnaporthe oryzae Mo*Pot2 gene in the inoculated leaves via qRT-PCR, and the levels were normalized to the expression level of the *Os*Ubi gene (*n*=3). (B) Phenotypes of HY, *flr2*, and *FLR2*-OE plants at 7 dpi after they were inoculated (by wounding) with the compatible *M. oryzae* isolate 70-15 (*n*=15 lesions). (C) Disease resistance of HY, *flr2*, and *FLR2*-OE plants in the blast nursery (*n*=3). Scale bar=20 cm. (A–C) The error bars correspond to 1 SD as determined by the duplicate analyses. The data are shown as the mean ±SD. The asterisks indicate significant differences from the control, as determined by one-way ANOVA followed by Tukey’s test (*P<0.05, **P<0.01, ***P<0.001). Three independent biological replicates of (A–C) were tested, each yielding similar results.

To further determine the function of *FLR2* in the response to rice blast disease, we created two *FLR2-*OE lines (*FLR2*-OE#4 and #7) by using a *GFP* fusion gene under the control of the *35S* promoter, and their up-regulated *FLR2* mRNA levels and protein levels were confirmed (Supplementary Fig. S6). The *flr2* (Li *et al.*, 2016), OE lines, and wild-type plants were inoculated with the compatible *M. oryzae* 70-15 isolate by spraying at the seedling stage (Fig. 4A, left panel). *flr2* exhibited increased blast resistance, as reflected by the significantly decreased lesion area and fungal biomass in the *flr2* mutant compared with the wild-type HY plants at 7 dpi (Fig. 4A, middle and right panels). *FLR2*-OE#4 and #7 exhibited increased plant susceptibility to fungal infection, as reflected by the increased lesion area and fungal biomass in the OE plants compared with the wild-type plants. We then inoculated the leaves (via the wound method) with the compatible *M. oryzae* isolate 70-15 (Fig. 4B). *flr2* developed 30–40% smaller and shorter lesions at the wound-inoculated sites than did HY (Fig. 4B, left and middle panel). The fungal biomass of *flr2* was reduced to 10–20% of that of HY (Fig. 4B, right panel). In contrast, *FLR2*-OE#4 and #7 displayed 30–40% longer lesions and greater fungal biomass than did HY (Fig. 4B, middle and right panel). To further determine the resistance of the transgenic rice, the materials were replanted in a blast nursery. As shown in Fig. 4C, compared with the wild-type plants, the *flr2* was more resistant to rice blast in the field, whereas the *FLR2*-overexpressing plants displayed highly susceptible phenotypes.

**Fig. 5.**
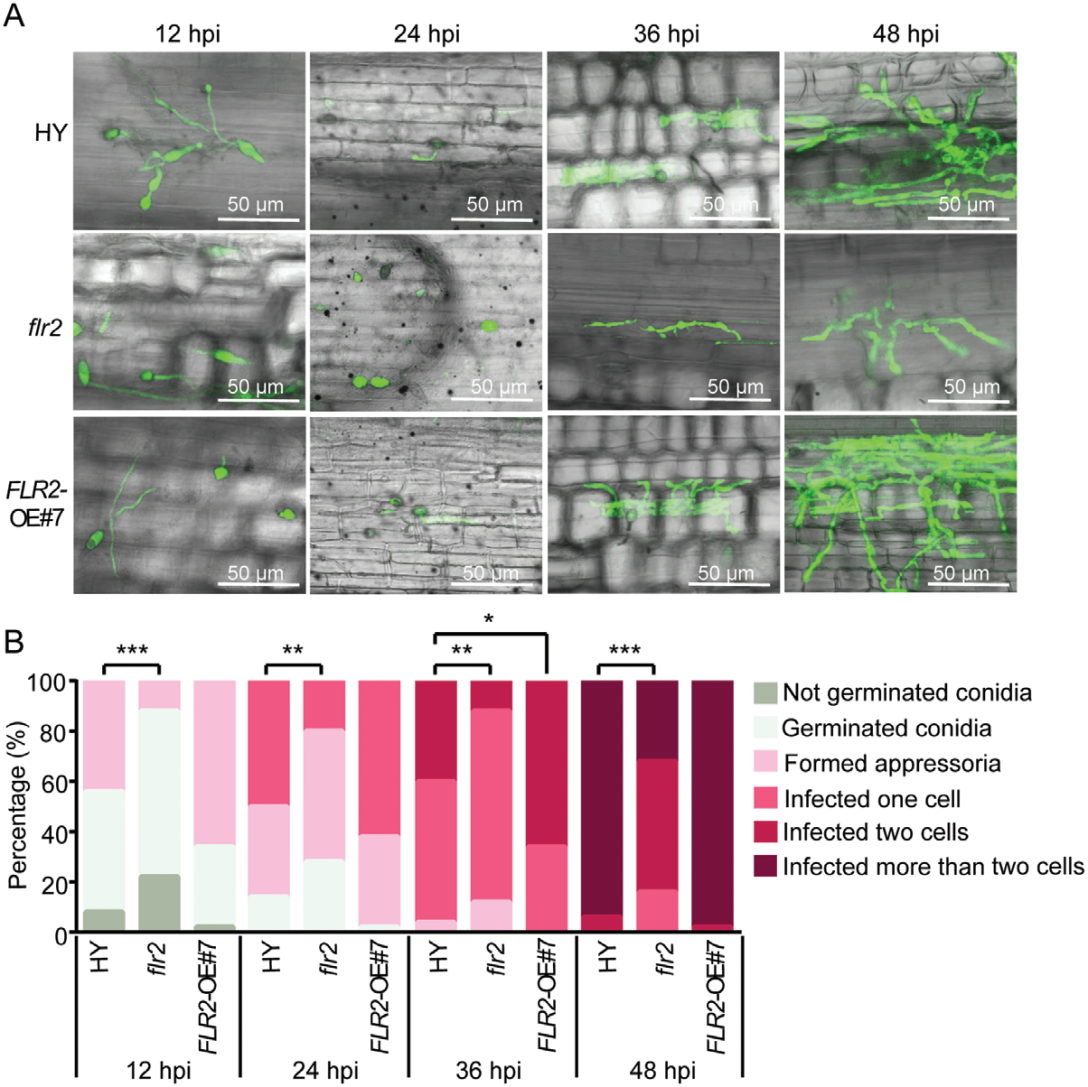
Fluorescence signals of GFP-tagged *Magnaporthe oryzae* strain 70-15GFP invasive hyphae in the leaf sheaths of *flr2*, *FLR2*-OE, and HY wild-type plants. (A) Confocal images showing the invasive *M. oryzae* hyphae in the epidermal cells of *flr2*, *FLR2*-OE#7, and HY leaf sheaths. Scale bar=50 μm. (B) Percentage of invasive hyphae growth at 12, 24, 36, and 48 hpi. The asterisks indicate significant differences from the control as determined by Crosstabs, followed by a χ ^2^ test (*P<0.05, **P<0.01, ***P<0.001). Fifty hyphae were counted per replication, and the experiment was repeated three times, each yielding similar results. (This figure is available in color at *JXB* online.)

We then investigated the invasive hyphal growth of the virulent isolate 70-15GFP in the leaf sheaths. We observed that more penetration sites formed appressoria inside the epidermal cells of the wild-type HY plants and *FLR2*-OE#7 lines than inside those of the *flr2* at 12 h post-inoculation (hpi). More invasive hyphae formed primary hyphae on *FLR2*-OE#7 lines than on wild-type HY plants at 24 hpi, which was opposite to the results observed for the *flr2*. The primary hyphae branched out into secondary invasive hyphae and extended to the neighboring cells at 36 hpi (Fig. 5A). Notably, the number of invasive hyphae in the *flr2* was significantly lower than that in the HY plants and *FLR2*-OE lines at 36 and 48 hpi (Fig. 5B).

The use of broad-spectrum resistant and robust resistant varieties represents an environmentally friendly and cost-effective method for controlling rice blast fungi. Dozens of broad-spectrum resistance genes have been reported in recent works (Azizi *et al.*, 2016; Deng *et al.*, 2017; Li *et al.*, 2017), providing information for breeding new disease-resistant varieties. To determine whether knocking out *FLR2* conferred broad-spectrum blast resistance to plants, we performed inoculation experiments with six blast isolates (HNB7, HNB52, HNB119, HNB145, HNB154, and Guy11); the HNB52 strain belongs to the ZC16 physiological race, and HNB7, HNB119, HNB145, and HNB154 belong to the ZB13 physiological race (Xing *et al.*, 2017). We found that the *flr2* was more resistant to these strains than were the HY plants, while the *FLR2*-OE lines were more susceptible to these strains than were the HY plants (Supplementary Fig. S7). Thus, the *FLR2* mutation has a certain degree of broad-spectrum resistance.

**Fig. 6.**
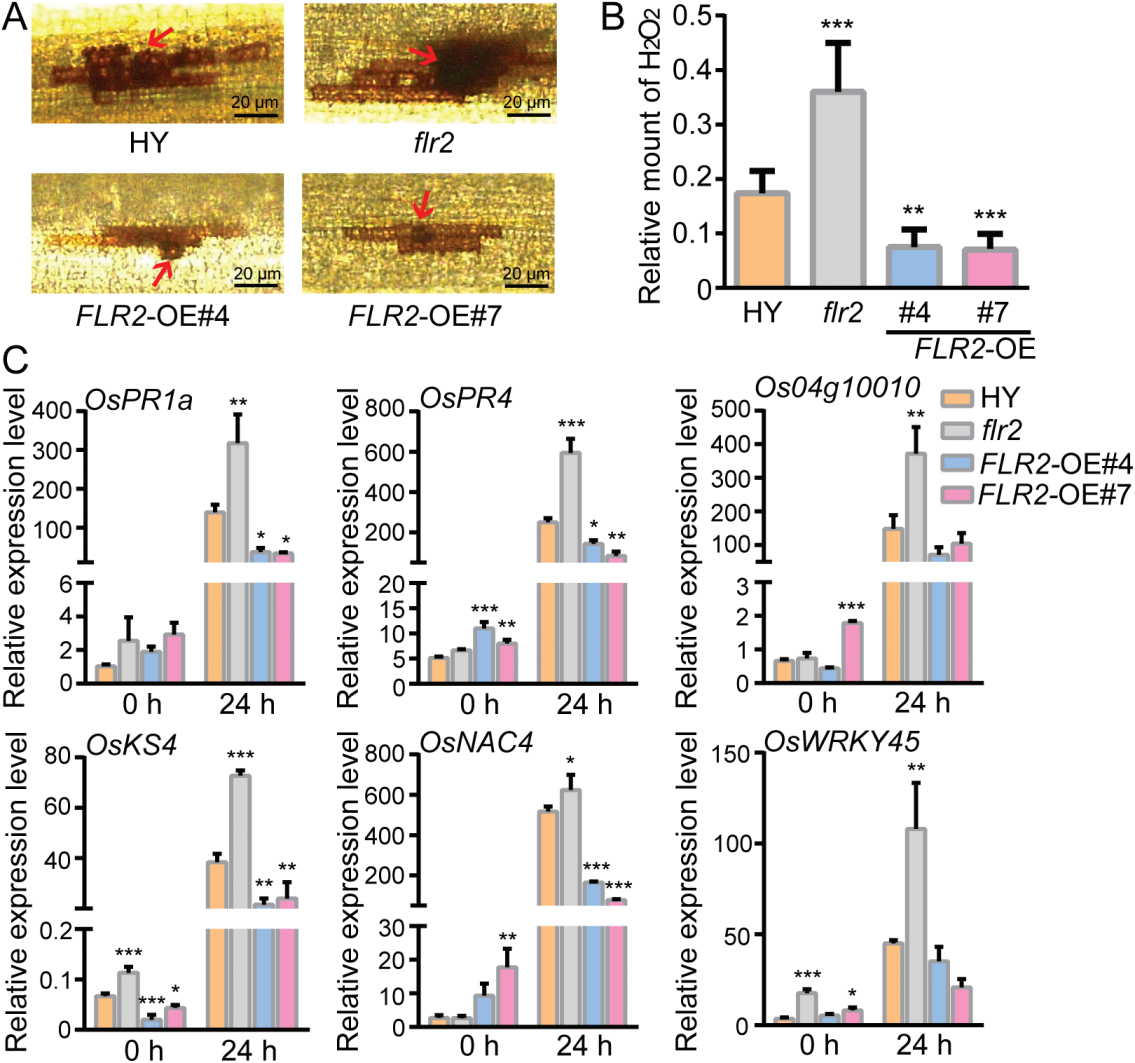
*FLR2* negatively regulates resistance to disease caused by *Magnaporthe oryzae*. (A) DAB staining of the infection sites in *flr2*, *FLR2*-OE, and HY wild-type plants at 3 dpi. The arrows indicate the infection structures of appressoria. Scale bar=20 μm. (B) Quantification of H_2_O_2_. The relative amount of H_2_O_2_ was calculated on the basis of the pixels of images via Photoshop with the following formula: H_2_O_2_ area per rectangle=pixels of H_2_O_2_ area per mycelial invasion site/pixels of the rectangle. (C) *M. oryzae*-induced expression of defense-related genes in 2-week-old transgenic rice lines and wild-type lines. The *OsActin* gene was used as an internal control, and relative expression levels were normalized to the level of the internal control at 0 h in the wild-type plants (*n*=3). The error bars correspond to 1 SD as determined by the duplicate analyses. The data are shown as the mean ±SD. The asterisks indicate significant differences from the control, as determined by one-way ANOVA followed by Tukey’s test (*P<0.05, **P<0.01, ***P<0.001). These experiments were performed three times, each yielding similar results. (This figure is available in color at *JXB* online.)

ROS signaling can modulate a broad range of biological processes involved in cell expansion, development, and responses to biotic and abiotic stimuli (Monshausen *et al.*, 2007; Baxter *et al.*, 2014; Duan *et al.*, 2014; Qi *et al.*, 2017). FER regulation has a profound role in the control of ROS in different biological processes (Wong *et al.*, 2007; Duan *et al.*, 2010; Li *et al.*, 2015); thus, H_2_O_2_ accumulation was tested in response to FLR2-mediated rice blast disease. DAB staining revealed that the *flr2* produced much greater amounts of H_2_O_2_ at the penetration sites of leaf cells than did the wild-type plants, while less H_2_O_2_ was produced in the leaf cells of the *FLR2*-OE lines in response to rice blast (Fig. 6A, B).

Upon pathogen attack, plants increase their immunity by activating sets of defense-related genes, including the salicylic acid (SA) signaling pathway marker gene *OsPR1a* (Agrawal *et al.*, 2000), the jasmonate (JA) signaling pathway marker gene *OsPR4* (Wang *et al.*, 2011), the dehydrogenase gene *OsO4g10010*, and the *kaurene synthase4* gene *OsKS4* (Agrawal *et al.*, 2000; Miyamoto *et al.*, 2016). Transcription factor genes such as *OsNAC4* and *OsWKRY45* also play roles as defense-related genes (Shimono *et al.*, 2007; Kaneda *et al.*, 2009). We therefore analyzed the expression of these defense-related genes in the transgenic lines and the wild-type plants at 0 h and 24 h after the leaves were inoculated with rice blast. The expression levels of the six defense-related genes were greater in *flr2* than in HY at 24 hpi. In contrast, the *FLR2*-OE lines had much lower expression levels of *OsPR1a*, *OsPR4, OsO4g10010*, *OsKS4*, *OsNAC4*, and *OsWKRY45* than did the HY plants (Fig. 6C). These results suggest that *FLR2*-mediated susceptibility was associated with decreased expression of defense-related genes induced by rice blast fungi.

### Growth traits related to the *FLR2/11* genes involved in response to *M. oryzae*

**Fig. 7.**
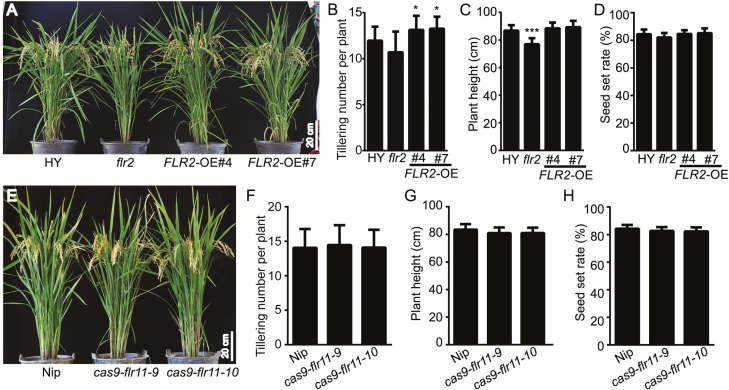
*FLR2/11* gene function in rice growth and development. (A) Morphology of mature *flr2*, *FLR2*-OE lines, and wild-type HY plants in normal field conditions. Scale bar=20 cm. (B–D) Tiller numbers, plant heights, and seed setting rates of *flr2*, *FLR2*-OE lines, and wild-type HY plants (*n*=25). (E) Morphology of mature *flr11* mutants and wild-type Nip plants. Scale bar=20 cm. (F–H) Tiller number, plant height, and seed setting rate of *flr11* mutants and wild-type Nip plants in normal field conditions (*n*=25). The error bars correspond to 1 SD as determined by the duplicate analyses. The data are shown as the mean ±SD. The asterisks indicate significant differences from the control, as determined by one-way ANOVA followed by Tukey’s test (*P<0.05, **P<0.01, ***P<0.001). Three independent biological replicates of (A–G) were tested, each yielding similar results. (This figure is available in color at *JXB* online.)

Compared with the wild-type plants, the *flr2* and *flr11* mutants were more resistant to rice blast (Fig. 3); therefore, we investigated whether the improved blast resistance would affect rice plant growth. At the mature stage, compared with those of the wild-type HY plants, the tiller numbers of the *flr2* was not significantly different, while the *FLR2*-OE lines had increased tiller numbers (Fig. 7A, B). Compared with that of wild-type HY, the plant heights of *flr2* were ~9% lower (Fig. 7A, C). FER functions in pollen tube reception and fertility in Arabidopsis (Escobar-Restrepo *et al.*, 2007). However, the *flr2* undergo normal pollen development (Li *et al.*, 2016), and the percentage of seed set did not differ between the *flr2* and wild-type HY plants (Fig. 7A, D). The number of tillers was similar between the *FLR11* mutants and the wild-type Nip plants (Fig. 7F). The seed setting rate and height did not obviously differ between the *flr11* mutants and the wild-type Nip plants (Fig. 7G, H). These results suggest that *flr2* and *flr11* exhibited increased disease resistance without significantly negative effects on their vegetative growth.

## Discussion

Members of the *Cr*RLK1L family have multiple functions and have been highly conserved throughout the evolution of plants (Lindner *et al.*, 2012; Liao *et al.*, 2017; Franck *et al.*, 2018). FER, a *Cr*RLK1L family member, is highly involved in most aspects of the plant life cycle, such as cell growth, root hair development, female fertility, and immunity (Lindner *et al.*, 2012; Kessler *et al.*, 2015; Franck *et al.*, 2018). The FER homologs FLR1 and FLR2 (Li *et al.*, 2016) controlled rice height, tillering, branching, and fructification to varying degrees. In the present study, we found that *Cr*RLK1L gene members were identiﬁed in *indica*, *japonica*, and *O. rufipogon* wild rice (Fig. 1), suggesting that *Cr*RLK1L is highly conserved during evolution. Moreover, expression analysis revealed that members of the *Cr*RLK1L/FLR family were ubiquitously expressed in different organs/stages in rice (Fig. 2C); this result might suggest its functional divergence. In addition, we systematically analyzed the functions of FLR family genes in rice resistance to *M. oryza*e (Fig. 3). The results showed that FLR2 and FLR11 are potential regulators involved in rice blast resistance without significant impact on growth. Our studies of FLRs can provide a reference for molecular breeding practices that can improve rice resistance and production through genetic manipulation.

The abundance of ROS and the pH of rice cells are two critical elements related to fungal pathogenicity. ROS bursts are a typical phenomenon in PTI and ETI pathways (Jones and Dangl, 2006; Couto and Zipfel, 2016). FER directly interacts with Rop-guanine exchange factors (RopGEFs), which then act as upstream regulators of RHO GTPases (RAC/ROPs). Activated RAC/ROPs further interact with several signal mediators, including NADPH oxidases, to regulate the production of ROS and plant immunity (Wong *et al.*, 2007; Duan *et al.*, 2010; Nibau and Cheung, 2011; Nagano *et al.*, 2016). FLR family members mediate rice immunity by possibly functioning as upstream regulators of Rac/ROP, which bind to the NADPH oxidase gene *OsRbohB* to regulate the production of ROS during rice blast infection (Wong *et al.*, 2007; Chen *et al.*, 2010; Kosami *et al.*, 2014; Nagano *et al.*, 2016). The possible mechanism is as follows: FLR may function as a PRR to detect microbial/PAMPs and may trigger ROS bursts, and certain effectors secreted by *M. oryzae* may directly or indirectly associate with FLR to suppress the production of ROS and evade plant immune defenses (Engelsdorf *et al.*, 2018).

Apoplastic pH is not only crucial for growth but is also important for immune responses in plants (Haruta *et al.*, 2014; Barbez *et al.*, 2017; Fernandes *et al.*, 2017). For pathogens, the ability to sense and adapt to or alter the pH of their environment is essential for their survival and pathogenicity (Fernandes *et al.*, 2017). During *M. oryzae* infection in rice and barley, once the infecting hyphae form and penetrate the plant epidermis, ammonia is rapidly released to stimulate apoplastic alkalinization through the PacC signaling pathway (Landraud *et al.*, 2013). Environmental alkalinization requires significant amounts of infected mycelia to release ammonia, but only a small portion of infected mycelia develops during the early stages of infection (Prusky and Yakoby, 2003; Fernandes *et al.*, 2017). To more rapidly infect the host and evade the host’s immune response, the pathogenic fungus *F. oxysporum* secretes a RALF1 homolog (F-RALF), which is involved in the FER-dependent signaling pathway to stimulate apoplastic alkalinization and suppress plant immunity to facilitate pathogenicity (Masachis *et al.*, 2016; Thynne *et al.*, 2017). Fungal RALF homologs are widely conserved among a diverse range of Ascomycota and Basidiomycota (Thynne *et al.*, 2017). Whether *M. oryzae* contains RALF homologs, which may interact with FLR to increase the extracellular pH, inhibit plant growth, and promote pathogenesis, is an open question. Therefore, determining whether RALF homologs exist in *M. oryzae* is warranted.

Both plant growth and immunity are related to several hormone signaling pathways, such as those of SA, JA, auxin, ABA, ethylene, and brassinosteroids (Ning *et al.*, 2017). In response to biotic stress, plants integrate multiple hormone signaling pathways to boost immunity, which can negatively affect plant development and growth, and ultimately affect grain yield. In Arabidopsis, FER is involved in the crosstalk between several hormone pathways that regulate cell growth, seed yield, and stress responses (Liao *et al.*, 2017; Franck *et al.*, 2018). In the present study, after plants were infected with the fungal strain 70-15, higher levels of *OsPR1a* (a marker gene of the SA pathway) and *OsPR4* (a marker gene of the JA pathway) were detected in *flr2* than in wild-type plants (Fig. 6C). Moreover, the *flr2* and *flr11 *mutants did not exhibit a significant reduction in growth (Fig. 7). Thus, as immune receptors, FLR gene family members (e.g. FLR2 and -11) have the potential to balance disease resistance with plant growth. The detailed molecular mechanisms of *FLR2-* and *FLR11-*mediated immune regulation still require further study. The *Cr*RLK1L/FLR family has been highly conserved throughout evolution (Franck *et al.*, 2018); thus, our work provides a reference for studying the role of the *Cr*RLK1L/FLR family in the function of immunity and growth in other crop species.

## Supplementary data

Supplementary material is available at *JXB* online

**Fig. S1.** Phylogenetic tree of *Cr*RLK1L family protein kinase domains from cultivated rice (*Oryza sativa japonica* and *O. sativa indica*), wild rice (*Oryza rufipogon*), Arabidopsis, *Physcomitrella patens*, and *Selaginella moellendorffii*.

**Fig. S2.** Conserved motif analyses. 

**Fig. S3.** Exon–intron structure of *FLR* genes.

**Fig. S4.** Chromosomal localization of *FLR* genes.

**Fig. S5.** Identification of *FLR* mutants. 

**Fig. S6.** Expression levels of *FLR2* in *FLR2*-OE lines, as detected by qRT-PCR and immunoblot analysis.

**Fig. S7.** Analysis of the resistance of the *FLR2* transgenic lines to different rice blast strains. 

**Table S1.** List of primers used in this research.

**Table S2.** Members of the FLR family.

erz541_suppl_Supplementary_Figures_S1-S7

erz541_suppl_Supplementary_Tables_S1-S2
