# Supplementary material for: Mutations of two FERONIA-like receptor genes enhance rice blast resistance without growth penalty
Source: J Exp Bot. 2020 Jan 27;71(6):2112–26. doi: 10.1093/jxb/erz541 (PMC7242082; doi:10.1093/jxb/erz541)
Supplement: erz541_suppl_Supplementary_Figures_S1-S7 [file erz541_suppl_supplementary_figures_s1-s7.pdf]

Supplementary data

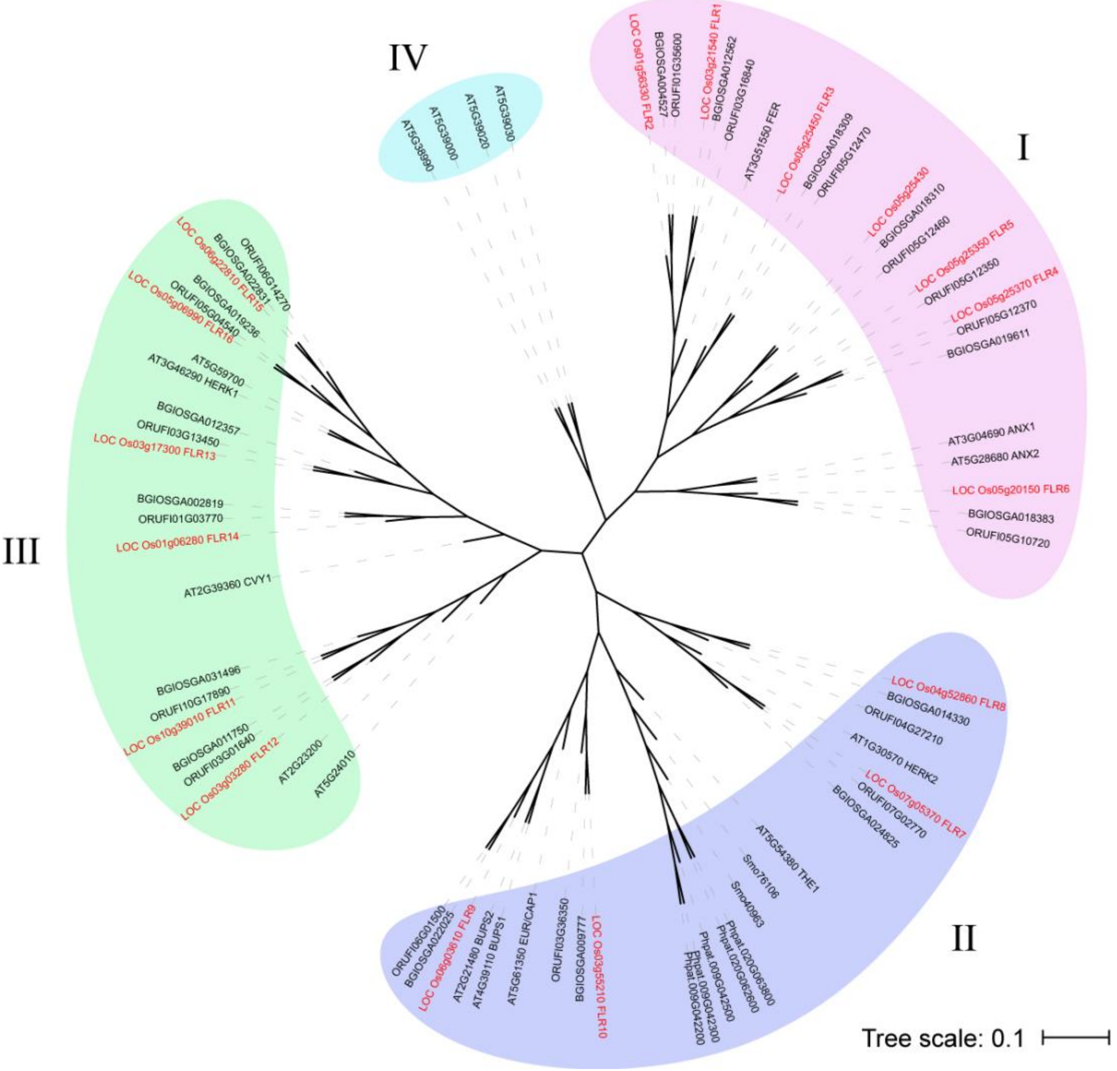

Fig. S1. Phylogenetic tree of *Cr*RLK1L family protein kinase domains from cultivated rice (*Oryza sativa japonica* and *O. sativa indica*), wild rice (*Oryza rufipogon*), *Arabidopsis*, *Physcomitrella patens* and *Selaginella moellendorffii*. The kinase domain sequences were subjected to the MEGA7 program to construct a phylogenetic tree via the neighbor-joining (NJ) method.

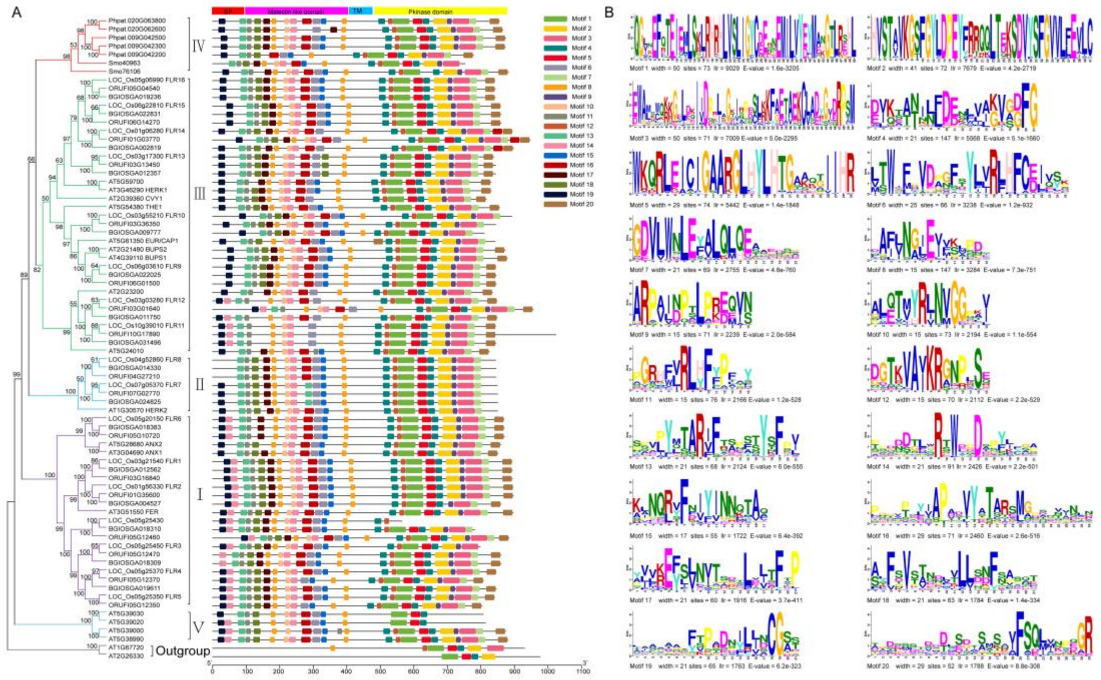

Fig. S2. Conserved motif analyses. (A) Motif analyses. Full-length protein sequences were subjected to the MEGA7 program to construct a phylogenetic tree via the NJ method. The motif composition analysis was performed by MEME (http://meme-suite.org/). Different motifs are shown in different-colored boxes. (B) Details of 20 motif sequences of *Cr*RLK1L proteins. Conserved motifs were identified within 74 *Cr*RLK1L proteins via MEME online software with ZOOPS models of minimum and maximum weights of 6 and 50 amino acid residues, respectively.

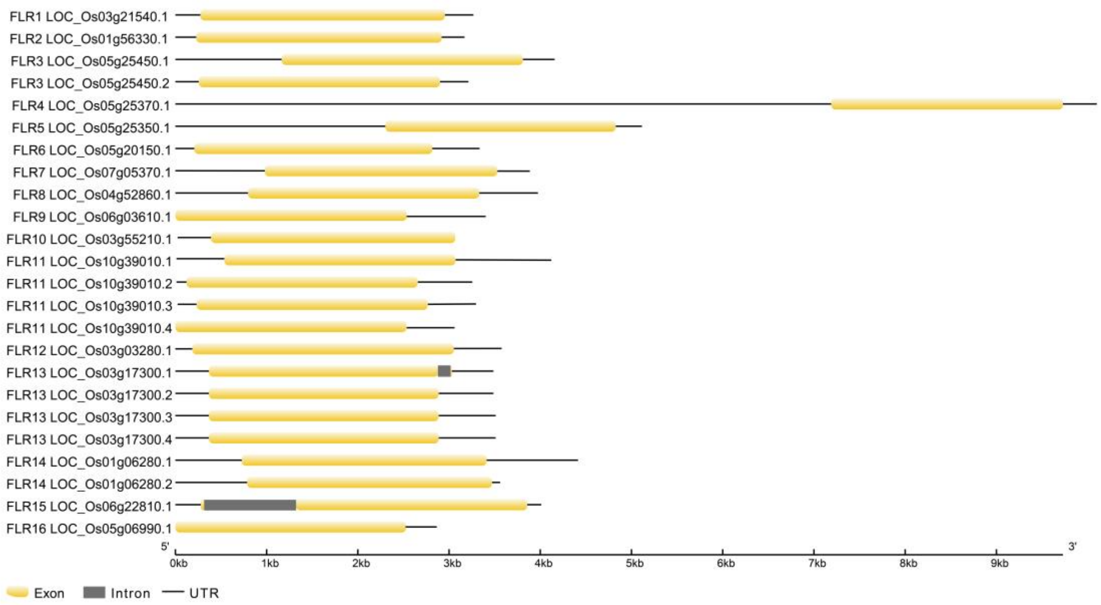

Fig. S3. Exon-intron structure of *FLR* genes. The black lines indicate untranslated 5'- and 3'-regions, the yellow boxes indicate exon, and the gray boxes indicate introns. The number after the rice locus identifier indicates the alternative splice form.

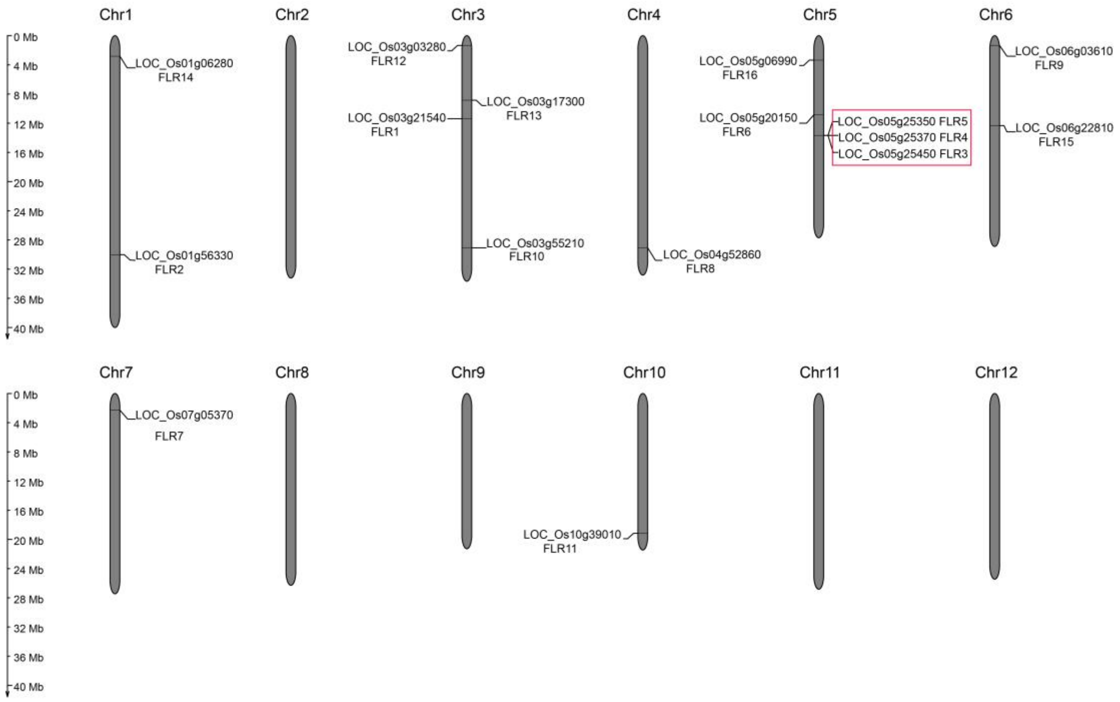

Fig. S4. Chromosomal localization of *FLR* genes. The rulers on the left indicate the length of the chromosome, and the gene cluster is framed by red lines within chromosome 5.

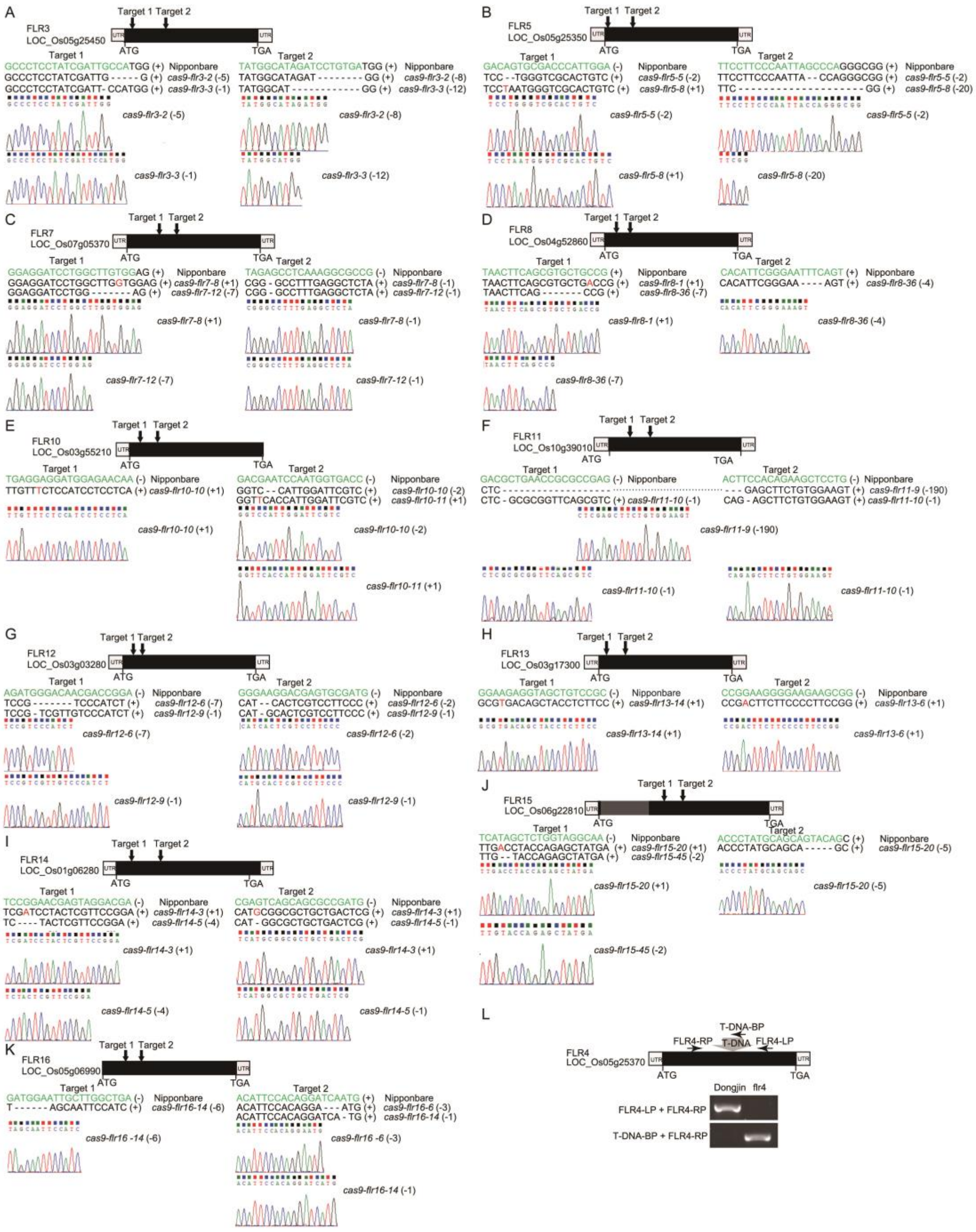

Fig. S5. Identification of *FLR* mutants. (A-K) Sequence alignment of the gRNA target region showing deleted or inserted bases within different *FLR* mutants. gRNA target sites are shown in green, and inserted bases are shown in red. (L) The *FLR4* T-DNA insertion mutant was identified by PCR amplification via three primers, as described by the Salk Institute (http://signal.salk.edu/cgibin/RiceGE). The exact sites for the T-DNA insertions (indicated by triangles) were mapped by PCR and DNA sequencing of

the PCR products. (A-K) ATG is the start codon, TGA is the stop codon, the black boxes are exons, the dark gray box is an intron, and the white boxes are untranslated 5'- and 3'-regions.

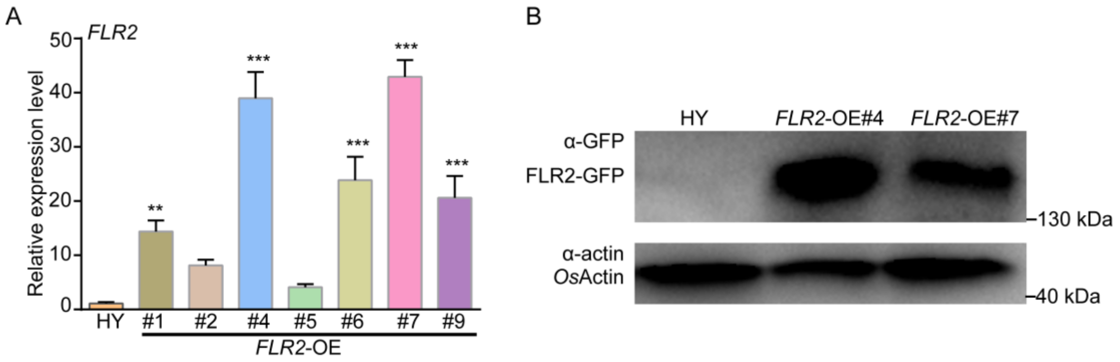

Fig. S6. Expression levels of *FLR2* in *FLR2*-OE lines, as detected by qRT-PCR and immunoblot analysis. (A) qRT-PCR analysis of *FLR2* mRNA levels in the HY and *FLR2*-OE lines. *OsActin* was used as a reference gene. Data represent the means±standard deviation (SD). The error bars correspond to one SD as determined by the duplicate analyses (n=3). The asterisks indicate significant differences as determined by one-way ANOVA followed by Tukey's test (* $P < 0.05$, ** $P < 0.01$, *** $P < 0.001$). (B) Immunoblot analysis FLR2 protein levels in the HY and *FLR2*-OE lines. An anti-GFP antibody was used to detect the FLR2 protein. OsActin served as a loading control. Three biological replicates of the experiment were tested, each yielding similar results.

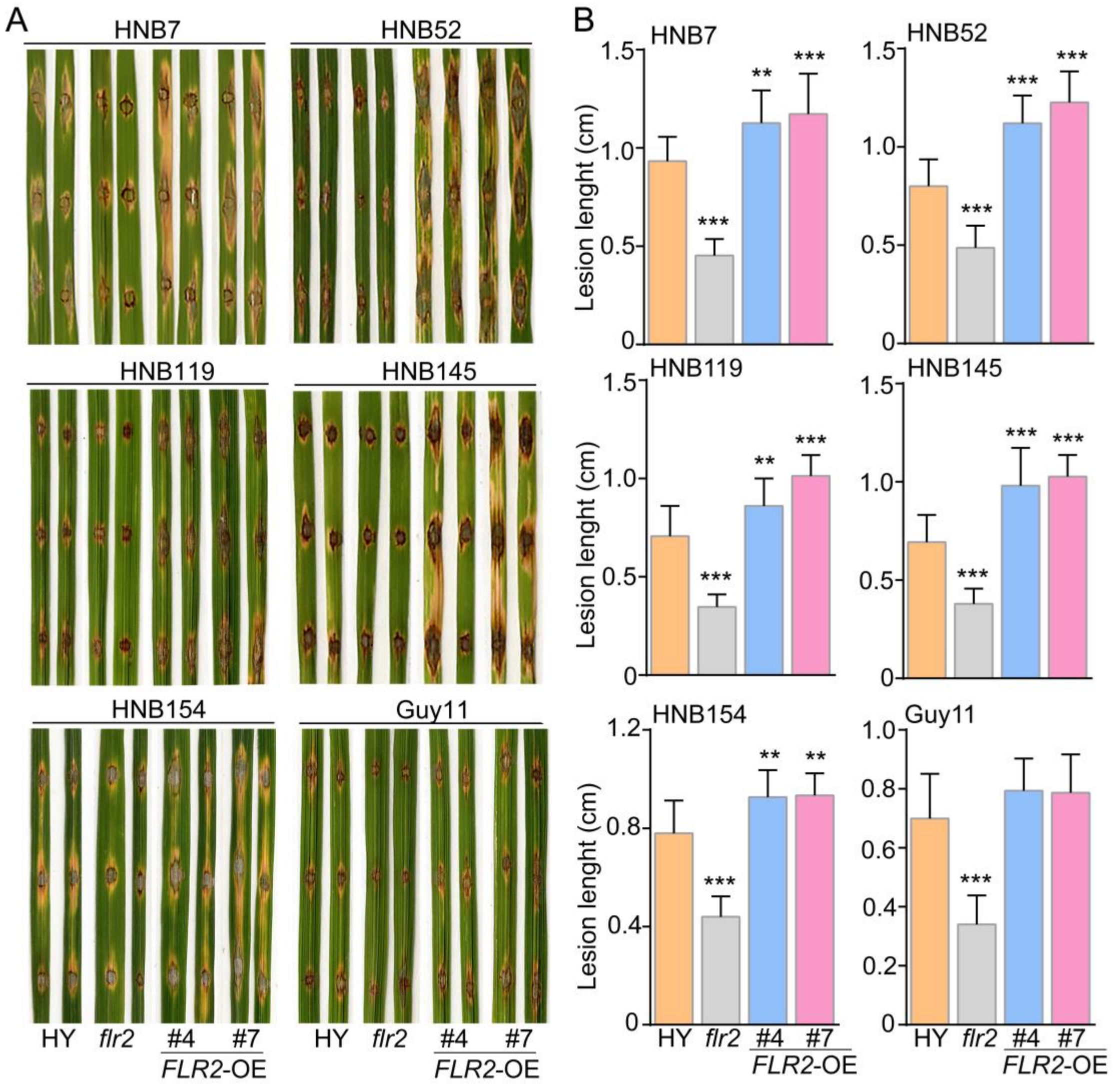

Fig. S7. Analysis of the resistance of the *FLR2* transgenic lines to different rice blast strains. (A) Phenotypes of HY, *flr2* and *FLR2*-OE plants inoculated with different rice blast strains. The blast strains HNB7, HNB52, HNB119, HNB145, HNB154 and Guy11 at a conidial concentration of 1×105/mL were used for wound inoculation. (B) Lesion length analysis of the inoculation sites (n=15). The error bars correspond to one SD as determined by the duplicate analyses. The asterisks indicate significant differences as determined by one-way ANOVA followed by Tukey's test (* P < 0.01, ** P < 0.01, *** P < 0.001). The experiments were performed three times, each yielding similar results.
