# Supplementary material for: Mutations of two FERONIA-like receptor genes enhance rice blast resistance without growth penalty
Source: J Exp Bot. 2020 Jan 27;71(6):2112–26. doi: 10.1093/jxb/erz541 (PMC7242082; doi:10.1093/jxb/erz541)
Supplement: erz541_suppl_Supplementary_Tables_S1-S2 [file erz541_suppl_supplementary_tables_s1-s2.pdf]

Table S1 Primers used in this study.

Primer Name	Primers(5'->3')	Source
**For CRISPR/Cas9**		
FLR3-Cas9-T1-F	CAGTGGTCTCAGGCATATGGCATAGATCCTGTGA	This paper
FLR3-Cas9-T1-R	CAGTGGTCTCAAAACTCACAGGATCTATGCCATA	This paper
FLR3-Cas9-T2-F	CAGTGGTCTCAGGCAGCCCTCCTATCGATTGCCA	This paper
FLR3-Cas9-T2-R	CAGTGGTCTCAAAACTGGCAATCGATAGGAGGGC	This paper
FLR5-Cas9-T1-F	CAGTGGTCTCAGGCAGACAGTGCGACCCATTGGA	This paper
FLR5-Cas9-T1-R	CAGTGGTCTCAAAACTCCAATGGGTCGCACTGTC	This paper
FLR5-Cas9-T2-F	CAGTGGTCTCAGGCATTCCTTCCCAATTAGCCCA	This paper
FLR5-Cas9-T2-R	CAGTGGTCTCAAAACTGGGCTAATTGGGAAGGAA	This paper
FLR7-Cas9-T1-F	CAGTGGTCTCAGGCAGGAGGATCCTGGCTTGTGG	This paper
FLR7-Cas9-T1-R	CAGTGGTCTCAAAACCCACAAGCCAGGATCCTCC	This paper
FLR7-Cas9-T2-F	CAGTGGTCTCAGGCATAGAGCCTCAAAGGCGCCG	This paper
FLR7-Cas9-T2-R	CAGTGGTCTCAAAACCGGCGCCTTTGAGGCTCTA	This paper
FLR8-Cas9-T1-F	CAGTGGTCTCAGGCATAACTTCAGCGTGCTGCCG	This paper
FLR8-Cas9-T1-R	CAGTGGTCTCAAAACCGGCAGCACGCTGAAGTTA	This paper
FLR8-Cas9-T2-F	CAGTGGTCTCAGGCACACATTCGGGAATTTCAGT	This paper
FLR8-Cas9-T2-R	CAGTGGTCTCAAAACACTGAAATTCCCGAATGTG	This paper
FLR9-Cas9-T1-F	CAGTGGTCTCAGGCACGCGGCGGAGCCCTTGAGC	This paper
FLR9-Cas9-T1-R	CAGTGGTCTCAAAACGCTCAAGGGCTCCGCCGCG	This paper
FLR9-Cas9-T2-F	CAGTGGTCTCAGGCATGTCTGTGATGAGCTCGTC	This paper
FLR9-Cas9-T2-R	CAGTGGTCTCAAAACGACGAGCTCATCACAGACA	This paper
FLR10-Cas9-T1-F	CAGTGGTCTCAGGCATGAGGAGGATGGAGAACAA	This paper
FLR10-Cas9-T1-R	CAGTGGTCTCAAAACTTGTTCTCCATCCTCCTCA	This paper
FLR10-Cas9-T2-F	CAGTGGTCTCAGGCAGACGAATCCAATGGTGACC	This paper
FLR10-Cas9-T2-R	CAGTGGTCTCAAAACGGTCACCATTGGATTCGTC	This paper
FLR11-Cas9-T1-F	CAGTGGTCTCAGGCAACTTCCACAGAAGCTCCTG	This paper
FLR11-Cas9-T1-R	CAGTGGTCTCAAAACCAGGAGCTTCTGTGGAAGT	This paper
FLR11-Cas9-T2-F	CAGTGGTCTCAGGCAGACGCTGAACCGCGCCGAG	This paper
FLR11-Cas9-T2-R	CAGTGGTCTCAAAACCTCGGCGCGGTTCAGCGTC	This paper
FLR12-Cas9-T1-F	CAGTGGTCTCAGGCAAGATGGGACAACGACCGGA	This paper
FLR12-Cas9-T1-R	CAGTGGTCTCAAAACTCCGGTCGTTGTCCCATCT	This paper
FLR12-Cas9-T2-F	CAGTGGTCTCAGGCAGGGAAGGACGAGTGCGATG	This paper
FLR12-Cas9-T2-R	CAGTGGTCTCAAAACCCATCGCACTCGTCCTTCCC	This paper
FLR13-Cas9-T1-F	CAGTGGTCTCAGGCAGGAAGAGGTAGCTGTCCGC	This paper
FLR13-Cas9-T1-R	CAGTGGTCTCAAAACGCGGACAGCTACCTCTTCC	This paper
FLR13-Cas9-T2-F	CAGTGGTCTCAGGCACCGGAAGGGGAAGAAGCGG	This paper
FLR13-Cas9-T2-R	CAGTGGTCTCAAAACCCGCTTCTTCCCCTTCCGG	This paper
FLR14-Cas9-T1-F	CAGTGGTCTCAGGCATCCGGAACGAGTAGGACGA	This paper
FLR14-Cas9-T1-R	CAGTGGTCTCAAAACTCGTCCTACTCGTTCCGGA	This paper
FLR14-Cas9-T2-F	CAGTGGTCTCAGGCACGAGTCAGCAGCGCCGATG	This paper

FLR14-Cas9-T2-R	CAGTGGTCTCAAAACCATCGGCGCTGCTGACTCG	This paper
FLR15-Cas9-T1-F	CAGTGGTCTCAGGCAACCCTATGCAGCAGTACAG	This paper
FLR15-Cas9-T1-R	CAGTGGTCTCAAAACCTGTACTGCTGCATAGGGT	This paper
FLR15-Cas9-T2-F	CAGTGGTCTCAGGCATCATAGCTCTGGTAGGCAA	This paper
FLR15-Cas9-T2-R	CAGTGGTCTCAAAACTTGCCTACCAGAGCTATGA	This paper
FLR16-Cas9-T1-F	CAGTGGTCTCAGGCAGATGGAATTGCTTGGCTGA	This paper
FLR16-Cas9-T1-R	CAGTGGTCTCAAAACTCAGCCAAGCAATTCCATC	This paper
FLR16-Cas9-T2-F	CAGTGGTCTCAGGCAACATTCCACAGGATCAATG	This paper
FLR16-Cas9-T2-R	CAGTGGTCTCAAAACCATTGATCCTGTGGAATGT	This paper
**Identification of homozygous**		
FLR3-Cas9-CK-F	TGCAAGTGTTCCCAACTCTTATAG	This paper
FLR3-Cas9-CK-R	ACATTACCATCTTTGGAGAAGGT	This paper
FLR5-Cas9-CK-F	TCGTTCCCACCTATCTTATAGCA	This paper
FLR5-Cas9-CK-R	GAATTCTCGAAGGATGTAGGC	This paper
FLR7-Cas9-CK-F	CTCCAGTAACTTGGAGATTGAG	This paper
FLR7-Cas9-CK-R	ACGTCATAGTTCTCAGCAGC	This paper
FLR8-Cas9-CK-F	CTTAAGCAGCCCTGGAATAGC	This paper
FLR8-Cas9-CK-R	GTGAGCATCACCTCAATTGCAT	This paper
FLR9-Cas9-CK-F	GCACCGACACGTCCTTCAC	This paper
FLR9-Cas9-CK-R	AGAACTCCGACTTCGACCTCAC	This paper
FLR10-Cas9-CK-F	GTTCGTCCCTGCATCTTCTGCTC	This paper
FLR10-Cas9-CK-R	TAAGGCTTGGAGGGGCTGAAAC	This paper
FLR11-Cas9-CK-F	GAGCTGTCCGGGAAGTCCG	This paper
FLR11-Cas9-CK-R	CATGTCACGTTCGACGTGGTC	This paper
FLR12-Cas9-CK-F	GCAAAGGGATAGCAAAGCT	This paper
FLR12-Cas9-CK-R	GTAACGGGAGGAGGAGGAG	This paper
FLR13-Cas9-CK-F	TGGGGCAACCTACTCTGATCAGC	This paper
FLR13-Cas9-CK-R	CACCTCGATGGCGTTGACGAAC	This paper
FLR14-Cas9-CK-F	ACAACTACCTCATCAGCTGC	This paper
FLR14-Cas9-CK-R	CTGGTCACTAGTCCATTCTCG	This paper
FLR15-Cas9-CK-F	GACAACTACCTGGTGGACTG	This paper
FLR15-Cas9-CK-R	GCGGTGTTGATCCTCCAC	This paper
FLR16-Cas9-CK-F	CAAACCGCAAGGATATTCACT	This paper
FLR16-Cas9-CK-R	CGCAGTATTGTAGACACTGTCTG	This paper
FLR4-LP	TCTTGAGGCGTTGCTTCCAT	This paper
FLR4-RP	TGGGGTCACCTTCAGCAAAG	This paper
T-DNA-BP	CCACAGTTTTCGCGATCCAGACTG	This paper
**Overexpression**		
FLR2-1300GFP-F	CGGTACCCGGGGATCCATGGGAAGCTCCAGATTCGTGCTCT	This paper
FLR2-1300GFP-R	CCATGTCGACTCTAGACCGTCCCTTGGGGTTCATGATCTGT	This paper
**qRT-PCR for gene expression**		
FLR1-RT-F	GGAAGATGACACGCCATACATACC	This paper
FLR1-RT-R	GGAGCCTCACGAGATACTGATAACC	This paper

FLR2-RT-F	TATCCAGATGTCCAGGCTAAAC	This paper
FLR2-RT-R	CAAGAACCACCAGACCTCCAAT	This paper
FLR3-RT-F	CTGAATTGTGGAGCCTCTGGTG	This paper
FLR3-RT-R	GGAGCGAAGGGTCTTGGTATGA	This paper
FLR4-RT-F	TATGCTTATCGCTTGTTTTTCT	This paper
FLR4-RT-R	TTTCGCCATTATTGTCTATTTC	This paper
FLR5-RT-F	AAAGCATTCTGGCAAGACTGAC	This paper
FLR5-RT-R	CCACCGTTGTACCATTATCTATCTC	This paper
FLR6-RT-F	AGATCGCACCTGACTGCCTGAA	This paper
FLR6-RT-R	CGCCGTTCTCAAATGTCTCCTG	This paper
FLR7-RT-F	ACTTGGTCCGGTTGCATTTC	This paper
FLR7-RT-R	ACTTGGTCCGGTTGCATTTC	This paper
FLR8-RT-F	TGAGGGTACTACAGTGGCAATC	This paper
FLR8-RT-R	TGGGAGGTCACTTCCATAAAG	This paper
FLR9-RT-F	GACAAGCTGAGCATCCAGGT	This paper
FLR9-RT-R	CTTGTACCACTTGACGGCCA	This paper
FLR10-RT-F	CTACTCAACCGCACAACAGAT	This paper
FLR10-RT-R	GACAAGTCGAGGTTAAGGACAC	This paper
FLR11-RT-F	AGCCGTACAGGTGGCTCCTCAGGGT	This paper
FLR11-RT-R	GGGGACTTCGACGACGCAAACATC	This paper
FLR12-RT-F	GCCTCCATTTCTTCCCTTTCAC	This paper
FLR12-RT-R	GGGCGTCTCCACAGCAGCTCCG	This paper
FLR13-RT-F	AGCAGAGGCTGGAGGCGTGCAT	This paper
FLR13-RT-R	GGTGCTGACGTGCGTCTTGTCC	This paper
FLR14-RT-F	CAGCTCAACCCTGCCGTGATG	This paper
FLR14-RT-R	CGGTGTTCGATTGTAAGAAAC	This paper
FLR15-RT-F	ATGGATACCGATTTGCCTTTAGC	This paper
FLR15-RT-R	TGTACGGAACTCATTGAGACCTTG	This paper
FLR16-RT-F	TGATAATTTCCCTCAGCCAAGC	This paper
FLR16-RT-R	CCAAGATACTGCCCAACACCCA	This paper
OsActin-RT-F	TGTATGCCAGTGGTCGTACCA	Yin *et al.*, 2018
OsActin-RT-R	CCAGCAAGGTCGAGACGAA	Yin *et al.*, 2018
OsPR1a-RT-F	GGAAGTACGGCGAGAACATC	Yin *et al.*, 2018
OsPR1a-RT-R	TGGTCGTACCACTGCTTCTC	Yin *et al.*, 2018
OsPR4-RT-F	AGTATGGATGGACCGCCTTCTGT	Yin *et al.*, 2018
OsPR4-RT-R	CTCGCAATTATTGTCGCACCTGTTC	Yin *et al.*, 2018
Os04g10010-RT-F	AAATGATTTGGGACCAGTCG	Li *et al.*, 2018
Os04g10010-RT-R	GATGGAATGTCCTCGCAAAC	Li *et al.*, 2018
OsNAC4-RT-F	TCCTGCCACCATTCTGAGATG	Li *et al.*, 2018
OsNAC4-RT-R	TTGCAGAATCATGCTTGCCAG	Li *et al.*, 2018
OsKS4-RT-F	TCGCATTGCGTGTGCAA	Li *et al.*, 2018
OsKS4-RT-R	TTGGAACTTCCGACATCGAAA	Li *et al.*, 2018
OsWRKY45-RT-F	CGGGTAAAACGATCGAAAGA	Jiang *et al.*, 2010

OsWRKY45-RT-R	GCTGAGACGACACATCAACAA	Jiang *et al.*, 2010
**qRT-PCR of rice gene for quantifiing *Magnaporthe oryzae***		
MoPot2-RT-F	ACGACCCGTCTTTACTTATTTGG	Li *et al.*, 2017
MoPot2-RT-R	AAGTAGCGTTGGTTTTGTTGGAT	Li *et al.*, 2017
OsUbi-RT-F	TTCTGGTCCTTCCACTTTCAG	Li *et al.*, 2017
OsUbi-RT-R	ACGATTGATTTAACCAGTCCATGA	Li *et al.*, 2017

Jiang CJ, Shimono M, Sugano S, Kojima M, Yazawa K, Yoshida R, Inoue H, Hayashi N, Sakakibara H, Takatsuji H. 2010. Abscisic acid interacts antagonistically with salicylic acid signaling pathway in rice-Magnaporthe grisea interaction. Molecular Plant-Microbe Interactions **23**, 791-798.

Li W, Zhu Z, Chern M, *et al.* 2017. A Natural Allele of a Transcription Factor in Rice Confers Broad-Spectrum Blast Resistance. Cell **170**, 114-126.e115.

Li Y, Zhang Y, Wang QX, *et al.* 2018. RESISTANCE TO POWDERY MILDEW8.1 boosts pattern-triggered immunity against multiple pathogens in Arabidopsis and rice. Plant Biotechnology Journal **16**, 428-441.

Yin X, Zou B, Hong X, *et al.* 2018. Rice copine genes OsBON1 and OsBON3 function as suppressors of broad-spectrum disease resistance. Plant Biotechnology Journal **16**, 1476-1487.

Table S2 Members of FLR gene family.

Name	MSU_ID	Indica ortholog	Wild rice ortholog	Arabidopsis ortholog	Cellular function in rice
FLR1	LOC_Os03g21540	BGIOSGA012562	ORUFI03G16840	AT3G51550 FER	FLR1 (DRUS1) and FLR2 (DRUS2) redundantly controlled rice height, tillering, branching, inflorescence, spikelet development, seed setting, floral organs and sugar utilization / conversion varying degrees (Li *et al.*, 2016; Pu *et al.*, 2017; Yang *et al.*, 2015)..
FLR2	LOC_Os01g56330	BGIOSGA004527	ORUFI01G35600	AT3G51550 FER	
FLR3	LOC_Os05g25450	BGIOSGA018309	ORUFI05G12470		
FLR4	LOC_Os05g25370	BGIOSGA019611	ORUFI05G12370		
FLR5	LOC_Os05g25350		ORUFI05G12350		
FLR6	LOC_Os05g20150	BGIOSGA018383	ORUFI05G10720	AT3G04690 ANX1, AT5G28680 ANX2	
FLR7	LOC_Os07g05370	BGIOSGA024825	ORUFI07G02770	AT1G30570 HERK2	
FLR8	LOC_Os04g52860	BGIOSGA014330	ORUFI04G27210	AT1G30570 HERK2	
FLR9	LOC_Os06g03610	BGIOSGA022025	ORUFI06G01500	AT4G39110 BUPS1, AT2G21480 BUPS2, AT5G61350 EUR/CAP1	Pollen tube growth and integrity (Liu *et al.*, 2016).
FLR10	LOC_Os03g55210	BGIOSGA009777	ORUFI03G36350	AT4G39110 BUPS1, AT2G21480 BUPS2, AT5G61350 EUR/CAP1	
FLR11	LOC_Os10g39010	BGIOSGA031496	ORUFI10G17890	AT2G23200, AT5G24010	
FLR12	LOC_Os03g03280	BGIOSGA011750	ORUFI03G01640	AT2G23200, AT5G24010	
FLR13	LOC_Os03g17300	BGIOSGA012357	ORUFI03G13450	AT2G39360 CVY1, AT5G59700, AT3G46290 HERK1	
FLR14	LOC_Os01g06280	BGIOSGA002819	ORUFI01G03770	AT2G39360 CVY1, AT5G59700, AT3G46290 HERK1	
FLR15	LOC_Os06g22810	BGIOSGA022831	ORUFI06G14270	AT2G39360 CVY1, AT5G59700, AT3G46290 HERK1	
FLR16	LOC_Os05g06990	BGIOSGA019236	ORUFI05G04540	AT2G39360 CVY1, AT5G59700, AT3G46290 HERK1	

Li C, Wang L, Cui Y, *et al.* 2016. Two FERONIA-like receptor (FLR) genes are required to maintain architecture, fertility, and seed yield in rice. Molecular Breeding **36**, 151.

Liu L, Zheng C, Kuang B, Wei L, Yan L, Wang T. 2016. Receptor-Like Kinase RUPO Interacts with Potassium Transporters to Regulate Pollen Tube Growth and Integrity in Rice. PLoS Genetics **12**, e1006085.

Pu CX, Han YF, Zhu S, *et al.* 2017. The Rice Receptor-Like Kinases DWARF AND RUNTISH SPIKELET1 and

2 Repress Cell Death and Affect Sugar Utilization during Reproductive Development. The Plant Cell **29**, 70-89.

Yang T, Wang L, Li C, Liu Y, Zhu S, Qi Y, Liu X, Lin Q, Luan S, Yu F. 2015. Receptor protein kinase FERONIA controls leaf starch accumulation by interacting with glyceraldehyde-3-phosphate dehydrogenase. Biochemical and Biophysical Research Communications **465**, 77-82.
